# Beyond Encapsulation: Inulin and Polydextrose as Dual-Function Carriers for Bioactive Preservation and Functional Enhancement of Elderflower (*Sambucus nigra* L.) Extract

**DOI:** 10.3390/pharmaceutics18101230

**Published:** 2026-09-28

**Authors:** Milica Radan, Anna Stasiłowicz-Krzemień, Zorana Mutavski, Jelena Mudrić, Ana Alimpić Aradski, Katarina Šavikin, Tatjana Stević, Dragana D. Božić, Smilja Marković, Judyta Cielecka-Piontek, Nada Ćujić Nikolić

**Affiliations:** 1 Institute for Medicinal Plant Research “Dr. Josif Pančić”, Tadeuša Koscuska 1, 11000 Belgrade, Serbia; zmutavski@mocbilja.rs (Z.M.); jmudric@mocbilja.rs (J.M.); ksavikin@mocbilja.rs (K.Š.); tstevic@mocbilja.rs (T.S.); 2 Department of Pharmacognosy and Biomaterials, Poznan University of Medical Sciences, Rokietnicka 3, 60-806 Poznan, Poland; astasilowicz@ump.edu.pl (A.S.-K.); jpiontek@ump.edu.pl (J.C.-P.); 3 Institute of Botany and Botanical Garden, “Jevremovac”, Faculty of Biology, University of Belgrade, Studentski trg 16, 11000 Belgrade, Serbia; alimpic.ana@bio.bg.ac.rs; 4 Department of Microbiology and Immunology, Faculty of Pharmacy, University of Belgrade, Vojvode Stepe 450, 11221 Belgrade, Serbia; dragana.bozic@pharmacy.bg.ac.rs; 5 Institute of Technical Sciences of Serbian Academy of Sciences and Arts, Knez Mihailova 35/IV, 11000 Belgrade, Serbia; smarkovic@itn.sanu.ac.rs

**Keywords:** elderberry flower extract, spray-drying, microencapsulation, physicochemical characterization, technological characterization, antioxidant activity, inhibition assays, antimicrobial activity, prebiotic activity, in vitro release study

## Abstract

**Background/Objectives**: Elderflower (*Sambucus nigra* L.) extract is a rich source of bioactive compounds with promising metabolic and gut health benefits. This study aimed to develop high-quality microencapsulates and evaluate the effects of inulin and polydextrose, used individually or in combination, on their stability, bioactivity, technological performance, and functional properties. **Methods**: Spray-drying was employed to produce four powder formulations: extract without a carrier addition, extract with 20% inulin or polydextrose addition, and extract with a combination of 10% inulin and 10% polydextrose. The resulting microparticles were characterized by their technological properties, such as powder yield, moisture content, bulk density, and tapped density. Fourier transform infrared spectroscopy, particle size distribution, and differential scanning calorimetry were used to define their physicochemical properties, while biological activities were evaluated using in vitro assays. **Results**: Carrier composition substantially influenced particle size, with inulin producing the smallest and most narrowly distributed particles. Incorporation of the extract into carbohydrate matrices promoted amorphous structure formation and enhanced thermal stability, while increasing rutin solubility by nearly 50-fold compared with its reported aqueous solubility. The extract’s biological activity was associated with its high polyphenol content, particularly chlorogenic acid and rutin, while microencapsulation effectively preserved the phenolic profile during storage. In addition to displaying potent DPPH (2,2-diphenyl-1-picrylhydrazyl) radical scavenging capacity and α-glucosidase inhibitory potential, the developed formulations exerted biological functionality on inhibiting Gram-positive bacteria and yeasts while fostering the growth of probiotic *Lactobacillus* strains. Formulations containing inulin, alone or combined with polydextrose, demonstrated the most promising overall biological potential. **Conclusions**: Inulin and polydextrose demonstrated dual functionality as carriers by protecting elderflower bioactives while enhancing their functional properties, highlighting the potential of the resulting microencapsulates as multifunctional ingredients for food, pharmaceutical, and nutraceutical applications.

## 1. Introduction

Growing health consciousness and the preference for natural solutions have significantly increased the demand for phytochemical-rich diets and functional foods, with global sales projected to reach $372.3 billion by 2032. Phytochemicals, through their anti-inflammatory, antioxidant, and gene-regulating activities, contribute to the prevention of chronic diseases, support immune function, and overall well-being.

In recent years, *Sambucus nigra* L. (Adoxaceae), also known as elderberry, has attracted considerable scientific and commercial interest due to its abundance of polyphenols and flavonoids. This deciduous shrub is widely distributed in sun-exposed habitats across Europe, Asia, North Africa, and North America. Both its dark purple berries, rich in anthocyanins and other phenolic compounds, and its aromatic white flowers, containing flavonols, phenolic acids, and essential oils, are traditionally used in food, beverages, and herbal medicine for their health-promoting properties [1]. Elderflowers are used to prepare infusions and extracts that are valued for their therapeutic benefits. Their bioactive constituents contribute to anti-inflammatory, antimicrobial, and mild diaphoretic effects, which support the relief of colds, coughs, and fever [2]. Owing to their polyphenol-rich composition, elderflowers have also demonstrated potential in the prevention of atherosclerosis and diabetes by attenuating inflammation and oxidative stress, enhancing pancreatic insulin secretion, and moderating postprandial glucose levels [2]. Among these constituents, rutin and chlorogenic acid serve as the primary bioactive compounds, offering numerous health benefits. Rutin exerts strong antioxidant, vasoprotective, and anti-inflammatory activities, while chlorogenic acid contributes significantly to glucose regulation, lipid metabolism management and structure of the gut microbiota [3,4].

However, the use of elderflower extracts in the nutraceutical and pharmaceutical industries presents several challenges, particularly regarding the stability and delivery of these key compounds. Chlorogenic acid is notably prone to degradation, exhibiting low thermal stability in the presence of water and a high susceptibility to isomerization and chemical destruction under high-temperature processing conditions [5]. Furthermore, the poor aqueous solubility of rutin severely limits its clinical application and oral bioavailability, representing a significant challenge in formulation development and drug delivery. [6]. Beyond individual compound vulnerabilities, the selection of appropriate extraction strategies to maximize bioactive yield, maintain extract stability and purity, and address cost-related factors remains critical for large-scale production and commercialization. Environmental and processing factors, including oxygen, light, enzymatic activity, extreme temperature, and pH conditions, can also alter the therapeutic properties of the extract, thereby limiting its overall stability. Therefore, the use of advanced delivery systems, such as biopolymer-based microencapsulates, is a promising strategy to enhance compound stability and bioavailability while enabling targeted, controlled release of bioactive compounds from elderflower extract, protecting them from environmental factors and improving their therapeutic efficacy in nutraceutical and pharmaceutical applications [7].

Microencapsulation using spray-drying technique is one of the most widely applied methods for producing stable powder formulations of plant extracts while preserving their functional and biological properties [8]. This method offers several advantages, including protection against environmental degradation, improved solubility and bioavailability, ease of handling and storage, and suitability for large-scale industrial production. The application of different carriers during the spray-drying process further enhances these benefits by improving powder properties, preventing stickiness, protecting sensitive bioactive compounds, and facilitating the drying process through encapsulation of the core material within a protective wall.

Inulin (IN) and polydextrose (PD) are polysaccharide-based carriers widely recognized for their effective stabilization and protective effects, owing to their biodegradable nature, structural flexibility that allows chemical modification, and capacity to deliver bioactives to specific sites. Inulin, a naturally occurring polysaccharide, serves not only as an encapsulating agent but also improves blood glucose and lipids, and acts as a prebiotic, stimulating the growth of beneficial gut microbiota [9]. Polydextrose, a synthetic polymer of glucose, is valued for its low caloric content, solubility, and ability to improve texture and stability in formulations, while also exerting prebiotic effects that support gut health and promote the production of beneficial short-chain fatty acids (SCFAs) [10]. Together, these carriers offer dual functionality by protecting sensitive bioactive compounds during processing and storage, while simultaneously contributing to the nutritional and functional value of the final product.

Previous studies on the encapsulation of *S. nigra* bioactives have predominantly investigated fruits, juices, or pomace while limited attention has been devoted to the stabilization and functionalization of phenolic compounds from elderflower extracts. Ribeiro et al. reported the preparation of spray-dried powders from elderberry extract (flowers, stems, and leaves) using carriers such as modified chitosan, sodium alginate, and gum arabic, demonstrating good stability and favorable morphological properties [11]. Likewise, spray-dried microencapsulates were developed using maltodextrin as a carrier, which, due to its high glycaemic index, is unsuitable for diabetic patients [12]. However, earlier studies provided limited insights into the detailed chemical characterization and further biological evaluation of the encapsulates.

Therefore, the present study focuses on the development of high-quality spray-dried powders of elderflower extract for use in natural, health-promoting products. The research was designed to investigate inulin and polydextrose as dual-function carriers for the spray-drying microencapsulation of elderflower (*S. nigra*) extract, both individually and in combination. Their selection is of particular relevance, as it is expected to not only protect and stabilize polyphenolic compounds but also potentially enhance the functional and health-promoting properties of elderflower microencapsulates, particularly with respect to metabolic regulation and gut health.

## 2. Materials and Methods

### 2.1. Chemicals

Inulin and polydextrose were provided by A.C.E.F. s.p.a. (Fiorenzuola d’Arda, Italy) and Thermo Fisher Scientific (Waltham, MA, USA), respectively. Standards of rutin and chlorogenic acid were obtained from Extrasynthese (Cedex, Genay, France). Acetonitrile (HPLC-grade) was acquired from Merck (Darmstadt, Germany). Milli-Q purification system (Millipore, Guyancourt, France) was used to prepare ultrapure water. Methanol was obtained from Chempur (Piekary Śląskie, Poland). Folin–Ciocalteu reagent, orthophosphoric acid, and sodium carbonate were supplied by Sigma-Aldrich Chemie GmbH (Munich, Germany). All reagents and enzyme preparations used in the α-amylase and α-glucosidase inhibition assays were purchased from Sigma-Aldrich (St. Louis, MO, USA). These included Lugol’s solution, acarbose, 4-nitrophenyl β-D-glucopyranoside (pNPG), dipotassium phosphate, potassium dihydrogen phosphate, sodium chloride, anhydrous sodium carbonate, sodium phosphate monobasic, sodium phosphate dibasic, α-glucosidase from *Saccharomyces cerevisiae* (type I), and α-amylase from porcine pancreas (type IV-B). Starch (1% solution) and hydrochloric acid were sourced from Carl Roth (Karlsruhe, Germany) and Zorka Pharma (Šabac, Serbia), respectively. The microbiological media, including Mueller–Hinton Broth (MHB), Tryptone Soya Broth (TSB), and Lactobacillus MRS (de Man, Rogosa and Sharpe) agar, were supplied by HiMedia (Mumbai, Maharashtra, India), whereas Sabouraud Dextrose Agar (SDA) was obtained from Thermo Scientific Oxoid (Pittsburgh, PA, USA).

### 2.2. Plant Material Extraction

*Sambuci flos* (*Sambucus nigra* L., Adoxaceae), an herbal material authorized in Poland as an over-the-counter medicinal product, was obtained from Zakład Zielarski KAWON-HURT (Gostyń, Poland; batch no. 1008.2023; EAN: 5909994335417). The extraction parameters optimized using a Box–Behnken experimental design, methanol concentration, extraction time, and the plant material-to-solvent ratio were adopted from a previous study [13]. Accordingly, 0.6 kg of plant material was extracted using 45% (*v*/*v*) methanol at a plant material-to-solvent ratio of 1:20 (*w*/*v*), for 15 min in 8 batches at 50 °C. Owing to the large amount of plant material processed, the procedure was proportionally scaled up, and a single extraction cycle was performed in an ultrasonic bath (Ulsonix Proclean 10.0S, Ulsonix, Zielona Góra, Poland; 40 kHz, 240 W). Following extraction, methanol was removed under reduced pressure using a rotary evaporator at 50 °C. The elderflower extract (EF) was then frozen at −22 °C and subsequently lyophilized for 72 h using a Heto PowerDry PL3000 freeze dryer (Thermo Fisher Scientific, Waltham, MA, USA). The instrument’s automatic program was applied to ensure complete water removal. After lyophilization, the dried extract was ground using a mortar. The extraction yield was calculated as the ratio of the dry extract mass obtained after lyophilization to the initial dry mass of the plant material used for extraction and expressed as a percentage (*w*/*w*).

### 2.3. Spray-Drying Process

The EF was subjected to spray drying either alone or in combination with carrier agents. For preparation of the feed solution, 133.7 g of freeze-dried EF was dissolved in 1000 mL of distilled water. Four formulations were prepared: (1) pure EF without a carrier (EF), (2) EF containing 20% inulin (EF + IN), (3) EF containing 20% polydextrose (EF + PD), and (4) EF containing a combination of carriers (10% IN and 10% PD) (EF + IN + PD). The carrier concentrations were calculated based on the dry weight of EF (*w*/*w*). Each carrier was dissolved separately in the EF solution, and the resulting mixtures were maintained at 40 °C under continuous magnetic stirring until the carriers were fully dissolved and homogeneous mixtures were obtained prior to spray drying.

The feed solutions were spray-dried using a Labtex ESDTi spray dryer (Labtex, Huddersfield, UK) equipped with a standard 0.5 mm nozzle. Drying process was executed at an inlet temperature of 145 ± 5 °C and an outlet temperature of 70 ± 5 °C. The drying air flow was maintained at 75 m^3^/h, with an atomization pressure of 3 bar and a feed solution rate of 10.5 mL/min and. All operating parameters were kept constant throughout the experiments.

Following spray drying, the resulting powders were recovered from the cyclone separator, placed in high-density glass containers, and kept at room temperature in a desiccator under dark conditions until subsequent analyses.

### 2.4. Technological Characterization of Spray-Dried Powders

#### 2.4.1. Powder Yield

The powder yield (PY) of the obtained elderflower microparticles was calculated according to Equation (1):

(1) $$PY\;\left(\%\right)=\;\frac{m_{p}}{m_{ep}}\times 100$$

The *m_p_* symbolizes the experimentally determined mass of powder recovered after drying, whereas *m_ep_* corresponds to the expected mass, calculated from the combined masses of the dried extract residue and the added carrier.

#### 2.4.2. Moisture Content

A halogen moisture analyzer HB43-s (Mettler Toledo, Columbus, OH, USA) was used to evaluate the moisture content (MC) of the EF microparticles by thermogravimetric analysis at 105 °C until a constant weight was reached. The calculation was performed according to Equation (2):

(2) $$MC\;\left(\%\right)=\;\frac{m_{m}}{m_{s}}\times 100$$

The *m_m_* and *m_s_* correspond to the mass of moisture present in the examined sample and the initial sample mass prior to spray-drying, respectively.

#### 2.4.3. Bulk Density, Tapped Density, Carr Index, and Hausner Ratio

The bulk density (*Q_bulk_*) of the EF microparticles was determined as described by Vidović et al. [14], with slight changes to the sample mass and graduated-cylinder volume due to the limited amount of powder available. Briefly, EF powder (1 g) was transferred to a glass cylinder (5 mL) and shaken at 300 rpm for 5 min. The resulting powder volume was then recorded, and the bulk density was calculated by dividing the powder mass by the measured volume (g/mL). Same cylinder was used to determine tapped density (*Q_tapped_*) after 120 manual tapping cycles, based on the final powder volume. The number of tappings was established from preliminary experimental observations, and 120 tappings were applied, after which no further appreciable change in powder volume was observed upon additional tapping. The Carr Index (CI) and Hausner Ratio (HR) were subsequently computed according to Equations (3) and (4), respectively, to characterize the flowability and cohesiveness of the powders.

(3) $$CI=\;\frac{Q_{tapped}\;∸\;Q_{bulk}}{Q_{tapped}}\times 100$$

(4) $$HR=\;\frac{Q_{tapped}}{Q_{bulk}}\;$$

Modified, literature-based procedures were adapted to the characteristics and limited quantity of the fine microparticles.

### 2.5. Physicochemical Characterization of Spray-Dried Powders

#### 2.5.1. Particle Size Distribution

The particle size distribution of the prepared powders was analyzed by laser diffraction using a Mastersizer 2000 instrument (Malvern Instruments, Worcestershire, UK) equipped with a Scirocco 2000 dry powder dispersion unit. The measurements were performed directly on the dried microparticles dispersion. Three independent measurements were performed for each powder formulation. Key parameters evaluated included *d_10_*, *d_50_*, and *d_90_*, denoting the particle diameters below which 10%, 50%, and 90% of the sample number and volume falls, respectively. The span value was calculated as a measure of the width of the particle size distribution according to Equation (5).

(5) $$Span=\;\frac{d_{90}∸\;d_{10}}{d_{50}}$$

Moreover, the surface-weighted mean diameter (D [3,2]) and volume-weighted mean diameter (D [4,3]) were determined, as well as the uniformity parameter. Uniformity parameter representing the instrument-derived measure of the complete particle size distribution, expressing the normalized absolute deviation of the individual size classes from the median particle size (*d_50_*), whereas lower uniformity values indicate a narrower particle size distribution.

The Fraunhofer model was used for particle-size calculation, with a particle refractive index of 0.000, absorption of 0, and dispersant refractive index of 1.000. The measurement size range was 0.020–2000 µm. The *d_10_*, *d_50_*, and *d_90_* particle-size percentiles, mean diameter, and span were recorded for each measurement and subsequently used for statistical analysis.

#### 2.5.2. Fourier Transform Infrared Spectroscopy

Structural properties of the powders and their respective carriers were evaluated with Fourier transform infrared (FTIR) spectroscopy in the attenuated total reflection (ATR) mode, providing insight into functional groups, structural features, and potential interactions between components. Nicolet iS10 spectrometer (Thermo Fisher Scientific, Stockholm, Sweden) was employed to record spectra over the range of 4000–400 cm^−1^, with a spectral resolution of 4 cm^−1^. Powders were analyzed in their solid state, and each sample was measured in duplicate.

#### 2.5.3. Differential Scanning Calorimetry

The spray-dried microparticles were examined for their thermal properties using a DSC131 Evo calorimeter from SETARAM Instrumentation, Caluire-et-Cuire, France. Approximately 5 mg of each sample was put into 30 µL aluminium pans that were hermetically sealed. Pan without sample served as a reference. Stabilization of the pants was performed at 20 °C for 5 min. Subsequently, they were heated at a rate of 10 °C/min to 350 °C, under a nitrogen flow of 20 mL/min. Data processing was performed with Calisto Processing 1.38 software (SETARAM Instrumentation), including baseline subtraction and determination of enthalpy values and changes (J/g).

### 2.6. Chemical Characterization of Spray-Dried Powders

#### 2.6.1. Total Phenolic Content

The prepared EF powders were evaluated for their total phenolic content (TPC) using a modified method with Folin–Ciocalteu (FC) reagent [15]. To a 200 µL aliquot of diluted EF, 800 µL of sodium carbonate solution and 1000 µL of FC reagent were added. Following the incubation period 2 h at room temperature, samples were spectrophotometrically analyzed at 765 nm against a blank. Measurements were performed in triplicate, and results were presented as mean values in milligrams of gallic acid equivalents per gram of dry weight (mg GAE/g DW).

#### 2.6.2. HPLC Analysis

Chromatographic analysis of the samples was performed using an Agilent 1260 RR HPLC system (Agilent, Waldbronn, Germany) with a diode-array detector (190–550 nm). The analysis followed the method described by Paczkowska-Walendowska et al. [16]. A LiChrospher RP-18 column (250 × 4.0 mm, 5 µm) utilizing a mobile phase consisting of solvent A (water containing 0.1% formic acid) and solvent B (acetonitrile) under gradient elution conditions: 2–20% B for first 35 min; 20–70% B for next 20 min; and 2% B for last 5 min. The temperature of column was kept at 30 °C. 1 mL/min was a flow rate, and 10 µL was set for the injection volume. Detection was carried out at 270 and 360 nm. Individual phenolic compounds in the microencapsulates (rutin and chlorogenic acid) were identified by comparing UV spectra and retention times with those of authentic standards. Quantification was achieved using calibration curves, and results were expressed as milligrams per gram of dry weight (mg/g DW).

### 2.7. Biological Evaluation of Spray-Dried Powders

#### 2.7.1. DPPH Radical-Scavenging Activity

The antioxidant capacity of the EF powders as well as reference standards was assessed with DPPH (2,2-diphenyl-1-picrylhydrazyl) assay [17]. Five dilutions of EF samples were prepared in methanol. Then, 2 mL was added to 0.5 mL of freshly prepared solution of DPPH in methanol (0.2 mg/mL). Methanol (2 mL) served as the blank. After incubating the mixtures for 30 min, at room temperature in the dark, the absorbance was measured against the blank at 517 nm using spectrophotometer. Equation (6) was used to calculate the radical-scavenging capacity (RSC), and the IC_50_ value, representing the concentration of extract required to neutralize 50% of DPPH radicals, was subsequently determined.

(6) $$RSC\;\left(\%\right)=\;\left[\frac{\left(A_{c}-A_{s}\right)}{A_{c}}\right]\times 100$$

Absorbance of the sample is denoted with $A_{s}$, whereas $A_{c}$ corresponds to the control absorbance. Ascorbic acid was employed as a reference standard.

#### 2.7.2. In Vitro α-Amylase and α-Glucosidase Inhibition Assays

Prepared EF powders (EF, EF + IN, EF + PD, and ED + IN + PD) were dissolved in 45% ethanol to obtain a stock concentration of 10 mg/mL. A series of twofold serial dilutions was subsequently prepared. Rutin was dissolved in methanol to reach 1 mg/mL, and further dilutions were as follows: 0.5, 0.25, 0.125, 0.0625, 0.03125, and 0.015625 mg/mL.

##### Alpha-Glucosidase Inhibition Assay

The potential to inhibit α-glucosidase enzyme was examined following the protocol described by Wan et al. [18]. The solution of α-glucosidase prepared in potassium phosphate buffer (0.1 M, pH 6.8) was added to 120 μL of the sample (S) to achieve 0.5 U/mL. After first incubation at 37 °C for 5 min, the 20 μL of substrate (pNPG, 5 mM) was added. Incubation was continued for the next 20 min at 37 °C. The reaction was terminated with the addition of 0.2 M sodium carbonate dissolved in potassium phosphate buffer (80 μL). Finally, by using a Multiskan Sky Thermo Scientific microplate reader (Vantaa, Finland), absorbance was detected at 405 nm. The buffer was used instead of the sample to prepare the control (C). To prevent the impact of sample color, the absorbance of the samples was adjusted by subtracting the extract color control (CC) absorbance, which contained the mixture of buffer and sample. Acarbose was used as the positive control. The results are expressed as mean ± standard error, after triplicate measurements. The percentage of inhibition of α-glucosidase activity was calculated using Equation (7):

(7) $$Inhibition\;(\%)\;=\;\frac{C\;-\;\left(S\;-\;CC\right)}{C}\;\times \;100\;$$

##### Alpha-Amylase Inhibition Assay

The procedure explained by Zengin et al. was used to evaluate the capability of EF powders and detected bioactive compounds to inhibit α-amylase enzyme [19]. Briefly, the mixture of sample (S) (25 μL) and sodium phosphate buffer (0.1 M, pH 6.8, with 6 mM sodium chloride) α-amylase solution (50 μL) was prepared to achieve a concentration of 0.5 mg/mL. The 10 min incubation at 37 °C was followed by the addition of starch solution (0.1%) (50 μL) and further for next 10 min. 1 M hydrochloric acid (25 μL) was used to terminate the reaction. For the visualization, Lugol’s solution was added (100 μL), and the absorbance was measured at 630 nm using same Multiskan as for the α-glucosidase assay. The buffer was used to replace enzyme and sample in the substrate control (SC) and enzyme control (EC), respectively. The CC was prepared same as for the α-glucosidase assay, and acarbose was also used as a positive control. All the measures were performed in triplicate, and results are expressed as mean ± standard error. The percentage inhibition of α-amylase activity was calculated using Equation (8):

(8) $$Inhibition\;(\%)\;=\;\frac{(S\;-\;CC)\;-\;EC)\;}{SC}\;\times \;100$$

#### 2.7.3. Antimicrobial Activity

The antimicrobial activity of the prepared EF powders was established by determining the minimum inhibitory concentration (MIC), minimum bactericidal concentration (MBC) and the minimum fungicidal concentration (MFC) using the broth microdilution method performed according to the recommendations of the Clinical and Laboratory Standards Institute (CLSI). MIC values for bacteria were determined according to CLSI M07 [20] and interpreted using CLSI M100 [21], 35th edition (2025). MIC values for yeasts and filamentous fungi were determined according to CLSI M27 (2024) [22]. The activity was tested against three Gram-positive bacteria: *Staphylococcus aureus* ATCC 29213, *Streptococcus pyogenes* Rosenbach ATCC 19615, and *Staphylococcus epidermidis* ATCC 12228; two Gram-negative bacteria: *Pseudomonas aeruginosa* ATCC 27853 and *Escherichia coli* ATCC 25922, and yeast *Candida albicans* ATCC 10231 (Thermo Scientific, Waltham, MA, USA).

The Müller–Hinton Agar (MHA) was used to sub-culture all bacterial strains, while Sabouraud Dextrose Agar (SDA) served for the yeast growing. For further preparation, Müller–Hinton Broth (MHB) and Triptone Soya Broth (TSB) were used to cultivate overnight bacterial strains and yeast, respectively. Inoculum turbidity was standardized to 0.5 McFarland (1 × 10^8^ CFU/mL for bacteria, 1 × 10^6^ CFU/mL for yeast) and further diluted in sterile saline to attain final concentrations of 1 × 10^6^ CFU/mL, and 2 × 10^4^ CFU/mL, respectively. Aliquots of the extract dilutions in suitable broth media (MHB for bacteria or TSB for yeast) were then dispensed into sterile 96-well polystyrene microtiter plates.

Specifically, the mixture of microbial suspension (20 μL) and resazurin solution (10 μL) as an indicator of growth, were added for a final volume of 200 µL per well. After incubation of plates containing bacteria at 37 °C for 24 h and for yeast at 25 °C for 3–4 days, MIC was determined. Microbial growth was indicated by the development of a pink coloration in the wells, resulting from the enzymatic reduction in purple resazurin to pink resorufin by metabolically active microbial cells. Depending on the type of microorganism, an appropriate volume of wells without indicated growth was inoculated in sterile liquid media or MH/SAB agar. After the incubation of 24 h at 37 °C and 3 days at 25 °C, the MBC and MFC were determined, respectively. All tests were carried out in triplicate. Positive controls consisted of microbial strains in suitable growth media supplemented with standard antibiotics (tetracycline for bacteria and fluconazole for yeast) and resazurin, whereas negative controls contained only the medium and indicator.

#### 2.7.4. Prebiotic Activity

The prebiotic activity of the tested EF powders was assessed using four probiotic Gram-positive bacterial strains (Thermo Scientific, USA: *Lactobacillus acidophilus* ATCC 314 (LA), *Lactobacillus casei* ATCC 393 (LC), *Lactobacillus gasseri* ATCC 19992 (LG), and *Lactobacillus plantarum* ATCC 8014 (LP)); two strains of the probiotic yeast *Saccharomyces cerevisiae* var. *boulardii* (i.e., *Saccharomyces boulardii*, SB); and a mixed probiotic culture comprising all strains (Mix-LA, LG, LP, LC, and SB). Microorganisms were incubated for 24–48 h at 35 °C under aerobic, anaerobic, or microaerophilic conditions (BD GasPak™ EZ CO_2_ Gas Generating Systems and Supplies: Pouch Systems, Fisher Scientific, USA) on Lactobacillus MRS (de Man, Rogosa and Sharpe) agar, and SDA, following standard cultivation procedures for probiotic microorganisms [23,24]. The effects of prepared samples on probiotic-growth stimulation were assessed according to previously described methodology [25], with minor modifications. For these assays, the extracts were suspended in MRS broth (Oxoid) or Sabouraud dextrose broth (Oxoid) and tested at concentrations ranging from 0.125 mg/mL to 2 mg/mL. Each concentration was dispensed in triplicate into a 96-well microtiter plate and inoculated with 5 × 10^5^ CFU/mL of the respective microorganisms. Following incubation, optical density was measured at 600 nm for bacterial cultures and 530 nm for yeast cultures using a microplate reader (Multiskan FC Microplate Photometer, Thermo Fisher Scientific, Waltham, MA, USA). The prebiotic effect was expressed as the percentage of microbial growth relative to the positive control, calculated as (OD_sample_/OD_positive control_) × 100.

OD_sample_—optical density of probiotic strains of microorganisms grown in the presence of the tested EF powders.

OD_positive controle_—optical density of probiotic strains of microorganisms grown only in medium.

### 2.8. Solubility Determination

An excess amount of prepared samples (EF, EF + IN, EF + PD, EF + IN + PD) was separately added to 5 mL of purified water in closed vials. The samples were continuously agitated at 250 rpm for 72 h at room temperature using an orbital shaker (Unimax 1010, Heidolph, Schwabach, Germany) to achieve equilibrium conditions. After that, the suspensions were filtered through membrane filters, and the concentration of dissolved rutin was quantified by HPLC method (Section 2.6.2). All measurements were conducted in triplicate, and the obtained results are reported as mean values ± standard deviation.

### 2.9. In Vitro Release Study

The release profiles of rutin and chlorogenic acid from spray-dried powders (EF, EF + PD, EF + IN, EF + PD + IN) were investigated under sink conditions in three media with pH values relevant for the gastrointestinal tract—pH 1.2 (0.1 M HCl), pH 4.5 (phosphate buffer), and pH 6.8 (phosphate buffer). The experiment was carried out using beakers placed in a water bath (Erweka DT70, Erweka, Langen, Germany) with a stirring speed of 90 rpm for a duration of 60 min at a controlled temperature of 37 ± 1 °C. Firstly, samples with a mass of about 50 mg were accurately weighed and placed in dissolution medium (10 mL). Small volumes of the dissolution medium (approximately 1 mL) were withdrawn and filtered at predefined time points (1, 5, 15, 30, and 60 min), with each sample immediately replaced by an equal volume of fresh medium. The concentrations of the dissolved marker compounds (rutin and chlorogenic acid) in the collected samples were analyzed using the HPLC method (Section 2.6.2). The study was conducted in triplicate, and the data are expressed as mean values ± standard deviation.

### 2.10. Stability Analysis

Spray-dried powders of EF were further evaluated for stability over a period of three months. Samples were stored under controlled conditions at 40 °C and 75% relative humidity, as well as at room temperature with exposure to daylight. The concentrations of key bioactive constituents, rutin and chlorogenic acid, were determined by HPLC (Section 2.6.2), while the total polyphenol content was assessed spectrophotometrically (Section 2.6.1).

### 2.11. Statistical Analysis

Technological and physicochemical data are expressed as the mean ± standard error (SE). Statistical evaluation was performed using one-way analysis of variance (ANOVA), followed by Tukey’s honestly significant difference (HSD) post hoc test in IBM SPSS Statistics version 16.0.

For the antimicrobial assays, a more conservative approach commonly adopted in antimicrobial susceptibility testing was applied. All measurements were conducted in triplicate, and the highest recorded value among the replicates was reported as the MIC and MBC/MFC. Consequently, these results are presented as single values rather than as mean ± standard deviation.

## 3. Results and Discussion

### 3.1. Extraction of Plant Material

The dry extract yield was 24.84% (*w*/*w*), which is within the range of values reported for ultrasound-assisted extraction (UAE) of *S. nigra* flowers using hydroethanolic solvents [26]. Comparative studies evaluating several extraction techniques (maceration, continuous agitation, microwave-assisted, ultrasound-assisted, and enzyme-assisted extraction) have shown that UAE consistently provides the highest recovery of phenolic compounds from elderberry plant material among the tested methods [27]. Similarly, direct comparisons between maceration, Soxhlet extraction, and UAE applied to other plant matrices have also shown that UAE outperforms conventional techniques, yielding markedly higher extraction efficiency under equivalent solvent systems [28].

### 3.2. Technological and Physicochemical Characterization of Spray-Dried Powders

#### 3.2.1. Powder Yield and Moisture Content

The powder yield varied considerably depending on the carrier composition. The addition of PD resulted in a pronounced increase in yield (60.91%) compared to the control sample (27.68%), indicating that PD significantly improved solid recovery during drying (Table 1). This effect is most likely associated with enhanced matrix formation and higher total solid content, which reduces material loss during the process [29]. The incorporation of IN alone led to a moderate increase in yield (32.84%), whereas the combination of IN and PD (50.26%) produced a yield lower than PD alone, suggesting possible interactions between the carriers that affected system viscosity or matrix structuring during drying. Similar trends have been reported in the literature. Marafon et al. investigated the spray drying of *Araucaria angustifolia* extracts using inulin and polydextrose as carriers [30]. They reported yields of 67.7% and 60.6%, respectively, confirming that both carriers can facilitate powder formation and improve solid recovery during drying. Lower process yields (approximately 25–41%) were reported by Ribeiro et al. for spray-dried *S. nigra* flower and leaf extracts encapsulated with modified chitosan, sodium alginate, or gum Arabic, highlighting the strong influence of carrier type on powder recovery [11]. Compared with those formulations, the use of PD in the present study resulted in substantially higher process yield, whereas the control and IN-containing formulations fell within or closer to the range reported by Ribeiro et al. [11].

Moisture content of powders ranged from 2.61% to 4.55%. The lowest moisture level was observed in the EF + IN sample (2.61%), indicating that IN contributed to improved drying efficiency and/or enhanced water binding within the powder matrix. In contrast, the addition of PD alone did not significantly reduce moisture content compared to the control, suggesting that its primary contribution was related to yield rather than residual water removal. The combination of IN and PD resulted in intermediate moisture values (3.33%), reflecting the combined but not fully synergistic effects of both carriers. These values were lower than those reported for spray-dried *S. nigra* flower extracts encapsulated with 30–78% maltodextrin (4.65–8.27%) [12], indicating efficient water removal under the applied drying conditions. Moreover, all powders exhibited moisture contents below 5%, which is generally considered desirable for spray-dried plant powders, as it contributes to improved microbiological stability and reduced deterioration during storage [31].

#### 3.2.2. Powder Flow and Compressibility Parameters

Powder density and flow characteristics are important indicators of the physical quality of spray-dried powders, as they influence powder handling, storage, transportation, and further processing. Bulk and tapped density provide information on particle packing behavior, while Carr’s index (CI) and Hausner ratio (HR) are commonly used to evaluate powder flowability and cohesiveness [32].

In the present study, bulk density values ranged from 0.280 to 0.305 g/mL, whereas tapped density values varied between 0.433 and 0.452 g/mL (Table 1). The highest bulk density was observed for EF + PD (0.305 g/mL), indicating the formation of more compact particles and/or improved particle packing efficiency, which may be related to differences in carrier composition and matrix-forming ability during spray drying. Conversely, EF + IN exhibited the lowest bulk density (0.280 g/mL), suggesting differences in particle morphology and packing behavior. The obtained density values are comparable with those reported for spray-dried plant extract powders containing carbohydrate-based carriers [33].

The CI values ranged from 32.52% to 37.50%, while HR values varied from 1.48 to 1.60. According to commonly applied powder flowability classifications, CI values above 32% and HR values above 1.45 indicate poor to very poor flowability, reflecting highly cohesive powder systems [32]. Accordingly, all formulations exhibited poor flow properties. EF + IN showed the highest CI (37.50%) and HR (1.60), indicating the highest cohesiveness and the poorest flowability among the tested powders. This behavior may be associated with the hygroscopic nature of inulin, which can promote moisture adsorption and increase interparticle cohesion, thereby reducing powder mobility.

Although PD markedly improved process yield and slightly increased bulk density, it did not substantially improve powder flowability, as EF + PD exhibited CI (32.52%) and HR (1.48) values comparable to the control formulation. Overall, carrier composition affected the technological properties of spray-dried microencapsulates, particularly yield and moisture content, whereas the improvement of flow properties was limited.

#### 3.2.3. Particle Size Distribution

Particle size distribution is an important quality attribute of spray-dried powders, since it can influence powder flowability, packing behavior of future formulation, dispersibility, reconstitution, and the performances of the resulting microparticulate system. In the present study, particle size was evaluated both on a number- and volume-weighted basis, with the corresponding d_10_, d_50_, d_90_, span, D [4,3], D [3,2], and uniformity parameters presented in Table 2 and Figure 1. The particle size distributions differed markedly depending on whether the results were expressed by number or by volume, which is expected for spray-dried powders containing a relatively small fraction of larger particles.

When expressed on a volume basis, the carrier-free spray-dried elderflower extract (EF) exhibited a d_50_ of 7.38 µm and a volume-weighted mean diameter D [4,3] of 19.40 µm, with d_10_ and d_90_ values of 2.36 and 35.51 µm, respectively. The addition of inulin markedly reduced the particle size, resulting in d_50_ and D [4,3] values of 5.11 and 5.40 µm, respectively. Moreover, the d_90_ decreased from 35.51 µm for EF to 9.45 µm for EF + IN, accompanied by a pronounced reduction in span from 4.49 to 1.52. These changes indicate a considerably narrower particle size distribution following incorporation of inulin. A similar, although less pronounced, effect was observed for the combined EF + IN + PD formulation, for which d_50_, d_90_, and D [4,3] were 6.20, 52.46, and 21.31 µm, respectively, with a span of 8.10. In contrast, EF + PD showed the broadest distribution, characterized by a d_90_ of 152.35 µm and a span of 19.02, together with the highest D [4,3] value (39.09 µm). Thus, although the median particle size of EF + PD (7.87 µm) was relatively close to that of the carrier-free extract, the markedly higher d_90_ and span indicate the presence of a substantial fraction of larger particles or agglomerates.

The differences among the formulations are particularly relevant when compared with the number-weighted distributions. On a number basis, all formulations exhibited very small d_50_ values (0.312–0.324 µm), whereas the corresponding volume-weighted d_50_ values were considerably higher (5.11–7.87 µm). This discrepancy indicates that a large number of fine particles coexist with a smaller number of substantially larger particles, which contribute disproportionately to the total powder volume. Such behavior is characteristic of polydisperse spray-dried systems and highlights the importance of considering both number- and volume-weighted distributions when interpreting the particle size of plant-extract powders [8].

The observed particle sizes are generally consistent with previously reported spray-dried plant extracts, although direct comparison should be made cautiously since particle size is strongly dependent on feed composition, carrier type and concentration, atomization conditions, and the measurement approach. In a study of spray-dried everlasting flower (*Helichrysum plicatum*) extract exhibited a mean particle diameter of 6.49 µm, while incorporation of β-cyclodextrin, hydroxypropyl-β-cyclodextrin, maltodextrin, or whey protein reduced the mean particle diameter to approximately 3.99–4.86 µm [7]. These findings demonstrate that the addition and nature of the carrier can substantially influence the size of spray-dried plant-extract particles, which is consistent with the pronounced effect of inulin observed in the present study. Similar observations in study by Kuzmanović Nedeljković et al. support the present finding that carrier composition can strongly affect the particle-size characteristics of spray-dried botanical extracts [34].

The obtained values are also comparable with those reported specifically for elderberry flowers and stems. Ribeiro et al. reported spray-dried elderberry extract microparticles prepared with modified chitosan, sodium alginate, and gum arabic, with particle sizes in the micrometer range, demonstrating that spray drying can produce fine microparticulate elderberry systems [11]. The authors showed that carrier composition affected the morphology and physicochemical characteristics of the resulting powders. Similarly, de Souza et al. developed spray-dried *S. nigra* flower microparticles using maltodextrin and reported a mean particle diameter of 2.83 ± 2.55 µm, with approximately 60% of the particles falling between 1 and 4 µm [12].

More recently, Gościniak et al. reported substantially broader particle-size distributions for spray-dried elderberry systems prepared with galactooligosaccharides and chitooligosaccharides as a carriers [31]. Depending on the carrier-to-extract ratio, d_50_ values ranged from approximately 5.1 to 14.5 µm, while d_90_ values extended from 35 to more than 240 µm. The authors emphasized that carrier type and formulation ratio strongly affected agglomeration and distribution width. These findings are particularly relevant to the present results, as the broad distribution observed for EF + PD, especially its high d_90_ and span values, suggesting the formation of a heterogeneous population containing larger particles or agglomerates.

Interestingly, particle-size distribution did not directly correspond to powder flowability. EF + IN exhibited the narrowest particle-size distribution, but showed the poorest flowability. In contrast, EF + PD had the broadest distribution, although it showed better flowability parameters. This indicates that flowability is influenced not only by particle size and distribution width, but also by factors such as the proportion of fine particles, particle morphology, surface characteristics, and interparticle cohesion. The high proportion of fine particles in EF + IN may have increased cohesive interactions and consequently impaired flow despite its narrow distribution.

The present results demonstrate that the carrier composition, rather than spray-drying conditions alone, substantially influenced the particle-size characteristics of elderflower powders. Under the constant spray-drying conditions applied in this study, inulin produced the smallest and most narrowly distributed particles, whereas polydextrose alone resulted in the broadest distribution. The combined inulin–polydextrose formulation exhibited intermediate behavior. The smaller and more uniform particle population obtained with inulin may be advantageous for powder handling and reconstitution, whereas the broad distribution observed for EF + PD should be considered when evaluating its technological performance.

#### 3.2.4. Fourier-Transform Infrared Analysis

FTIR spectroscopy is a widely applied analytical technique for the chemical and structural characterization of microencapsulated delivery systems, providing valuable information on the presence of functional groups and the nature of molecular interactions. In this study, FTIR was employed to investigate spray-dried powders of elderflower extract, with the aim of assessing their chemical compatibility with the encapsulating agents, inulin and polydextrose. Figure 2 presents the FTIR spectra of the spray-dried EF, the pure carriers (inulin and polydextrose), and the microencapsulates produced using inulin, polydextrose, as well as their combination.

All elderflower microencapsulates, as well as the carriers, exhibited a wide absorption band between 3100 and 3500 cm^−1^, corresponding to O–H stretching vibrations [34]. Notable spectral differences were observed between the pure EF powder and the carrier-based samples. The pure spray-dried EF showed a peak at 3198 cm^−1^, while the encapsulates shifted to higher wavenumbers, 3220 cm^−1^ (EF + IN) and 3261 cm^−1^ (EF + PD and EF + IN + PD), indicating hydrogen bond formation between hydroxyl groups of the carriers and phenolic compounds. Inulin and polydextrose also exhibited absorption bands at higher wavenumbers, 3272 and 3291 cm^−1^, respectively. The reduced intensity of bands in the carrier-based powders further supports the entrapment of phenolics within the matrices [35]. Considering the structural differences in the carriers, with polydextrose being a highly branched glucose polymer with diverse glycosidic bonds, and inulin a linear β(2→1)-linked fructose polymer, the lower intensity observed for EF + PD may indicate a greater ability of polydextrose to interact with hydroxyl groups of the extract’s bioactive compounds.

An additional band at 2933 cm^−1^ was detected in all samples, corresponding to C–H stretching vibrations of alkyl groups [34]. The fingerprint region between 1500 and 1600 cm^−1^ was evident in all EF samples and was attributed to C=C aromatic stretching of polyphenols, whereas the bands around 1300 cm^−1^ corresponded to OH bending of phenolic groups [36]. Absorption bands near 1000 cm^−1^ reflected C-H and C-O stretching vibrations, originating from both phenolic aromatic compounds and the carbohydrate backbone of the biopolymers. Notably, the microencapsulates with carriers showed reduced intensity and a shift in these peaks to a lower wavenumber (1032 cm^−1^) compared to the pure extract (1043 cm^−1^), further suggesting interactions between bioactives and carriers as well as alterations in glycosidic bonds [37].

Overall, the obtained spectra enabled the identification of characteristic functional groups and provided evidence of molecular interactions between elderflower polyphenols and carrier matrices. Such spectral shifts and intensity changes are of particular importance, as they indicate the establishment of intermolecular hydrogen bonding and the successful encapsulation of bioactives, which directly influence the stability, protection, and functional performance of microencapsulated systems.

#### 3.2.5. Thermal Characteristics of Spray-Dried Powders

DSC analysis was employed to investigate the influence of spray-drying encapsulation on the thermal behavior of the elderflower extract and carrier matrices, providing insight into thermal transitions, physical state modifications, and possible interactions between bioactive compounds and the carriers. DSC thermograms of spray-dried EF and corresponding microencapsulates prepared with inulin (EF + IN), polydextrose (EF + PD), and their combination (EF + PD + IN) are presented in Figure 3, while the thermal transition parameters are summarized in Table 3.

Polyphenol-rich plant extracts commonly exhibit multiple thermal transitions associated with water release, disruption of intermolecular hydrogen bonding, and progressive structural reorganization of phenolic-rich domains rather than a single degradation event [7,34,38]. Following the spray drying process, all encapsulated samples displayed markedly different thermograms.

The spray-dried EF exhibited two pronounced endothermic transitions. The first major thermal event centered at 157.86 °C (ΔH = 74.08 J/g) can be associated with the removal of residual bound water and low-molecular-weight volatile compounds, as well as early thermal transitions of amorphous bioactive constituents, which are commonly observed in spray-dried polyphenol-rich extracts [39]. A second and minor thermal event occurs at 196.25 °C with considerably lower enthalpy (4.82 J/g). The disappearance of the highly energetic thermal transition characteristic of the spray-dried extract and the emergence of two lower-energy thermal events may indicate structural rearrangement and increased amorphization of the extract matrix during spray drying, resulting from molecular redistribution and altered intermolecular interactions among its native constituents [38,40].

Encapsulation in carriers induced significant changes in the thermal profile compared with the pure spray-dried EF, including alterations in the position and enthalpy of thermal events.

Compared with the pure spray-dried EF, which exhibited a major thermal event at 157.86 °C, the EF + IN encapsulate showed a shifted thermal transition at 123.49 °C. Inulin-related encapsulation substantially reduced the intensity of endothermic transitions by a three-fold reduction in enthalpy, decreasing the first transition from 74.08 to 24.27 J/g. Lower enthalpy indicates that less energy is required for molecular reorganization, suggesting that the amorphous network has already undergone substantial structural relaxation and achieved a more stable configuration [41,42]. The second thermal transition of EF + IN remained centered at approximately 158 °C, although with markedly lower enthalpy. The reduced enthalpy and altered thermal profile suggest changes in the molecular organization and physical state of extract constituents after incorporation into the inulin matrix. These effects may be related to intermolecular interactions between inulin hydroxyl groups and phenolic compounds, as well as reduced molecular mobility and modified water association within the polysaccharide matrix [43,44]. The ability of inulin to adopt semi-crystalline or amorphous structures depending on processing conditions may contribute to the observed changes in thermal behavior [43,44].

A distinct thermal profile was observed for the polydextrose-containing spray-dried sample. The first thermal transition became almost negligible (ΔH = 4.74 J/g), while the second transition became dominant (ΔH = 30.57 J/g) and occurred at 144.41 °C. This behavior may be related to the highly branched and randomly polymerized glucose structure of polydextrose, which exhibits predominantly amorphous characteristics and strong glass-forming ability [38,45]. The redistribution of thermal events suggested that polydextrose promoted a different molecular organization of the extract constituents compared with pure EF, consistent with the ability of amorphous carbohydrate matrices to stabilize thermolabile bioactive compounds during drying and storage [46]. This is consistent with previous reports describing polydextrose as an effective stabilizing carrier for thermolabile bioactives [47].

The EF incorporated into the combined inulin–polydextrose matrix (EF + IN + PD) exhibited the most balanced thermal profile among the encapsulated systems. Although the first thermal transition occurred at the lowest temperature (120.55 °C), the second transition showed the highest enthalpy change (33.86 J/g) among encapsulates. The higher enthalpy associated with this transition should not be interpreted as reduced thermal stability; rather, it may reflect a greater extent of structural rearrangement within the dual-polysaccharide matrix. The distinct structural characteristics of inulin and polydextrose, combining a linear fructan polymer with a highly branched amorphous glucose-based polymer, polydextrose, may contribute to the formation of a more homogeneous amorphous matrix with reduced molecular mobility [38,43]. The DSC results indicate that the dual-polysaccharide system could provide a distinct thermal environment compared with single-polysaccharide formulations, suggesting complementary effects of inulin and polydextrose on the physical organization of the dried extract matrix. Similar effects have been reported for polysaccharide-based systems, where amorphous matrix formation and intermolecular interactions contribute to improved stabilization of polyphenolic compounds [48].

DSC results indicate that incorporation of EF into carbohydrate matrices probably promoting amorphous structure formation and enhanced thermal stabilization through carrier–bioactive interactions. These findings are consistent with reported effects of polysaccharide-based systems, where inulin and polydextrose contribute to the formation of protective amorphous matrices for bioactive compounds [43]. The redistribution of thermal events after spray drying, together with the reduction in the enthalpy transition characteristic of the native spray-dried EF, indicates successful molecular dispersion of elderflower phytochemicals within the carrier matrices.

### 3.3. Phytochemical Analysis and Stability of Spray-Dried Powders

The biological effects of elderflower extracts are largely attributed to their high polyphenol content, which is well recognized for its potential to support human health and enhance overall well-being. In this study, the TPC of the prepared microparticles of elderflower extracts was determined spectrophotometrically, with values ranging from 103.54 mg GAE/g to 124.46 mg GAE/g of dry extract (Table 4). Phenolic content was highest in the pure EF, followed by closely comparable concentrations in microencapsulates prepared with inulin (EF + IN), polydextrose (EF + PD), and their blend (EF + IN + PD). A similar trend was observed for the dominant individual bioactive compounds, rutin and chlorogenic acid. Rutin showed slightly higher values, ranging from 36.34 to 44.10 mg/g, whereas chlorogenic acid was present in amounts between 31.43 and 38.75 mg/g.

As anticipated, the incorporation of carriers at a proportion of 20% resulted in a moderate decrease in both TPC and individual phenolic contents, primarily due to the dilution effect. Nevertheless, the use of carriers proved beneficial for technological performance of the powders, which is a critical factor for their practical application. Notably, no statistically significant differences were detected among the samples containing different carriers, suggesting that inulin, polydextrose, and their mixture exhibit a comparable influence on the preservation of phenolic compounds during the microencapsulation process.

Considering the susceptibility of phenolic compounds to environmental stresses, which may lead to biotransformation or degradation during storage, a stability assessment of the prepared elderflower encapsulates was conducted. The study specifically evaluated the ability of the polysaccharide-based carriers, inulin and polydextrose, to protect the major bioactive compounds, rutin and chlorogenic acid, under different storage conditions (daylight at room temperature and 40 °C with 75% relative humidity). After three months of storage, no statistically significant changes were observed in the levels of total polyphenols or individual compounds across all formulations when compared with their initial values (Figure 4). These findings demonstrate that the use of inulin and polydextrose as carriers effectively preserves the phenolic profile of elderflower extracts, thereby maintaining their functional and biological potential during storage and supporting their application in functional foods and nutraceutical formulations.

Previous studies on the spray drying of elderflower extract provide limited data on the chemical composition of the resulting microencapsulates. Ribeiro et al. quantified only the TPC of encapsulated extracts, reporting values between 465 and 498 mg GAE/L [11]. Analyzing elderberry particles after 8 months of storage, the same authors observed an increase in TPC, which was attributed to oxidation and degradation processes leading to the formation of new phenolic-like compounds. Similarly, de Souza et al. developed spray-dried microparticles of *S. nigra* flower extract using maltodextrin and primarily evaluated flavonoid recovery, process parameters, and storage stability, rather than chemical profile of individual phenolic constituents [12]. In another study, Studzińska-Sroka et al. analyzed water–methanol extracts of different black elderberry flower cultivars and confirmed that rutin was the predominant bioactive compound, followed by chlorogenic acid [13]. The authors reported significantly lower TPC values than those obtained for the EF samples in the present study, with the Black Beauty cultivar exhibiting a TPC of 44.97 ± 1.15 mg GAE/g DW. This cultivar also showed the highest chlorogenic acid content among the investigated cultivars, reaching 23.73 ± 0.01 mg/g DW. Similarly, the rutin content reported for the Haschberg cultivar (38.37 ± 0.01 mg/g DW) was lower than that determined in the EF sample in the present study. Furthermore, Gościniak et al. reported TPC values of 57.75 ± 3.32 mg GAE/g DW for the Samyl 1 elderberry fruit cultivar, which were also significantly lower than those determined for the flower microparticles developed in the present study [31].

### 3.4. Biological Evaluation

#### 3.4.1. Radical Scavenging Activity

Oxidative stress occurs when the balance of free radical processes is disturbed, leading to elevated ROS levels that can disrupt vital biological functions. Antioxidants have attracted considerable interest for their protective roles in the body and in mitigating oxidative stress-related pathologies. Among natural sources, phenolics and flavonoids are recognized as the primary bioactive compounds responsible for health-promoting effects, whose antioxidant activity is largely due to free hydroxyl groups that effectively scavenge free radicals [49].

The antioxidant potential of the elderflower microparticles was assessed by measuring the change in the optical density of DPPH radicals, from which ${\mathrm{IC}}_{50}$ values were calculated (Table 5). The results indicated that the highest antioxidant capacity was observed for the pure EF powder and the powder prepared with inulin as a carrier (EF + IN), with no statistically significant difference between them. Slightly higher IC_50_ values were obtained for samples prepared with polydextrose (EF + PD) and with the combination of inulin and polydextrose (EF + IN + PD). Previous studies on elderflower extracts have also demonstrated their strong potential to reduce the DPPH radical, a property largely attributed to their high content of phenolic compounds such as chlorogenic acid and rutin [13]. Studzińska-Sroka et al. reported promising antioxidant activity of Black Beauty and Obelisk cultivars, with IC_50_ values of 53.15 mg Trolox equivalents (TE)/g and 49.92 mg TE/g, respectively [13]. Furthermore, Crişan et al. demonstrated that both geographical origin and extraction method can significantly influence the antioxidant capacity of elderflower extracts. In the DPPH assay, the highest antioxidant activity was observed for the extract obtained using ethanol-based UAE from samples collected in Mărișel, reaching 410.82 ± 19.69 mg TE/g [50]. As shown in Table 5, both chlorogenic acid and rutin exhibited marked radical scavenging activity in the DPPH assay, confirming that these phenolics are major contributors to the antioxidant capacity of elderflower extract and highlighting their importance as bioactive markers for functional formulations.

#### 3.4.2. α-Amylase and α-Glucosidase Inhibitory Potential of Spray-Dried Microparticles

Hypoglycemic effects of polyphenol-rich flower extract from *S. nigra* have been previously documented in several studies, highlighting their potential as dietary adjuncts for the management of diabetes and metabolic disorders [13,51]. Bioactive components from flowers of *S. nigra* L. increase glucose uptake in primary porcine myotube cultures and reduce fat accumulation in *Caenorhabditis elegans*. Postprandial glucose regulation is a key aspect of glycemic control and is closely associated with the development of diabetic complications and cardiovascular risk. Inhibition of carbohydrate-hydrolyzing enzymes, particularly α-amylase and α-glucosidase, represents an effective strategy to attenuate postprandial hyperglycemia, as these enzymes mediate the sequential breakdown of starch into absorbable monosaccharides.

The results presented herein demonstrate that studied samples exhibit a significantly higher inhibitory potential against α-glucosidase compared to α-amylase (Table 5). These findings are consistent with previous studies on elderflower hydroalcoholic extracts, which also showed superior α-glucosidase inhibition [13]. The strongest α-glucosidase inhibition was observed for powders prepared with inulin and for the combination of inulin and polydextrose, with no statistically significant difference between them. Slightly higher IC_50_ values were observed for the pure EF and for the powder prepared with polydextrose alone, suggesting that the presence of inulin may enhance enzyme inhibition, as previously reported for inulin-type fructooligosaccharides [52]. The potential inhibitory effects of the spray-dried EF can be attributed to the high content of rutin and chlorogenic acid, which demonstrated stronger α-glucosidase inhibitory activity than acarbose. Both compounds also exhibited notable α-amylase inhibition, with rutin being more potent. However, the elderflower microparticles themselves showed minimal α-amylase inhibitory activity, with the highest inhibition of 5.43% observed for the 10 mg/mL encapsulate prepared with inulin. Similar findings were reported by Studzińska-Sroka et al., who also demonstrated that the bioactive compounds in elderflower extracts, particularly rutin and chlorogenic acid, exhibited greater α-glucosidase inhibitory activity than the reference standard [13]. Among the investigated cultivars, Black Beauty showed the highest inhibitory potential (55.89%), followed by Black Tower (51.39%), Samyl (50.96%), and Obelisk (49.61%). In a study published by Crişan et al., ethanol-UAE extract exhibited significantly greater inhibitory activity than aqueous extracts and those obtained by infusion, with IC_50_ values of 2726.69 ± 395.33 μg/mL for α-glucosidase and 773.90 ± 4.59 μg/mL for α-amylase inhibition [50].

#### 3.4.3. Antimicrobial Activity of Spray-Dried Microparticles

The antimicrobial activity of elderflower extract has been extensively documented, with numerous studies demonstrating its effectiveness against a broad spectrum of microbial pathogens. Notably, elderflower extracts have often exhibited superior antibacterial activity compared to extracts derived from other parts of the plant, highlighting the flowers as a particularly rich source of antimicrobial compounds [53].

In agreement with previous studies, our results demonstrated that prepared spray-dried powders exhibit notable antimicrobial activity against all tested bacterial strains and yeast. The lowest MIC and MBC values were observed against common skin and soft tissue infection pathogens, namely *S. aureus* and *S. epidermidis*. These Gram-positive bacteria, together with *S. pyogenes*, were inhibited at relatively low extract concentrations, with MIC values below 1 mg/mL and MBC values below 1.5 mg/mL for pure extract (Table 6). Slightly higher MIC and MBC values were observed for the microencapsulated formulations containing carrier materials, which can likely be attributed to a dilution effect resulting from the incorporation of the carriers and the consequent reduction in the concentration of active compounds.

The pronounced antimicrobial activity of elderflower extract may be associated with its rich phenolic composition. Previous studies have demonstrated that elderflower phenolics are effective against clinically relevant and resistant pathogens, including methicillin-resistant *S. aureus* (MRSA), a major causative agent of skin and soft tissue infections such as furuncles, folliculitis, impetigo, and infected wounds [54]. Furthermore, Ferreira-Santos et al. reported promising antimicrobial activity of elderflower extracts against staphylococcal species, particularly *S. aureus* and *S. epidermidis* [55].

The results also demonstrated high inhibitory activity of the extracts against the yeast *C. albicans*, with the pure EF exhibiting MIC and MFC values of 0.7 mg/mL and 1.4 mg/mL, respectively. In addition, the extracts exhibited notable antimicrobial activity against the tested Gram-negative bacteria, *E. coli* and *P. aeruginosa* (Table 6). Consistent with previous reports, Gram-positive bacteria were generally more susceptible to elderflower extracts than Gram-negative strains [55]. This difference in sensitivity may be explained by the mode of action of polyphenolic compounds, which can disrupt microbial cells through membrane penetration and plasma membrane acidification. Gram-positive bacteria are more vulnerable to these effects because they lack the outer membrane characteristic of Gram-negative bacteria, which serves as an additional permeability barrier [56]. Accordingly, *P. aeruginosa*, a Gram-negative bacterium known for its intrinsic resistance mechanisms, was identified as the least susceptible microorganism among those tested.

As shown in Table 6, better antimicrobial activity was observed for the pure EF. Among the microencapsulated formulations, the extract combined with inulin exhibited the highest antimicrobial efficacy, followed by the formulation containing both inulin and polydextrose. The formulation prepared with polydextrose alone showed the lowest antimicrobial activity; however, it still demonstrated considerable inhibitory effects against all tested microorganisms.

The antimicrobial and antifungal activities observed in the present study further supports the potential application of elderflower microencapsulates as natural antimicrobial agents in pharmaceutical, cosmetic, and functional food formulations.

#### 3.4.4. Prebiotic Activity of Spray-Dried Microparticles

The tested elderflower extract and its formulations with IN and/or PD promoted growth of probiotic microorganisms in a concentration-dependent manner, primarily affecting *Lactobacillus* spp. and the mixed probiotic culture, while showing no noticeable effect on *S. boulardii* strains under these experimental conditions (Table 7, Figure 5).

The spray-dried elderflower extract (EF) alone exerted a moderate growth-promoting effect on all four *Lactobacillus strains*, with stimulation ranging from 123.7 ± 14.2% to 133.1 ± 15.3% at 2 mg/mL, while the mixed probiotic culture reached 137.2 ± 15.8%. Growth stimulation gradually decreased as the extract concentration declined, approaching control values at 0.125 mg/mL.

Incorporation of inulin enhanced the prebiotic activity of the formulation. EF + IN exhibited the highest stimulatory effect among the tested formulations, producing growth stimulation of 159.2–174.2% for individual *Lactobacillus* strains and 166.2 ± 19.9% for the mixed culture at 2 mg/mL. The formulation containing both inulin and polydextrose (EF + IN + PD) showed comparable activity, with growth stimulation ranging from 159.6 ± 19.2% to 177.7 ± 21.3%, although it did not consistently outperform EF + IN. In contrast, the EF + PD formulation produced only moderate stimulation, with values between 128.8 ± 14.8% and 138.2 ± 15.9% for *Lactobacillus* spp. and a maximum of 148.3 ± 17.1% for the mixed culture at the highest concentration.

Regardless of the formulation tested, both *S. boulardii* strains showed minimal response, with growth remaining between 95% and 104% of the control across the tested concentration range. The mixed probiotic culture consistently exhibited greater stimulation than the individual bacterial strains, suggesting more efficient utilization of the tested formulations in the multispecies culture (Table 7, Figure 5).

Evaluation of the carrier materials alone demonstrated that pure inulin produced substantially greater growth stimulation of all *Lactobacillus* strains and the mixed probiotic culture (at 2 mg/mL 169–185%, and 219, respectively) than polydextrose, which elicited only a modest response (approximately 100–114%). These findings are consistent with the higher effects of the inulin-containing formulations presented in Table 7.

The observed concentration-dependent stimulation of *Lactobacillus* spp. indicates that the elderflower formulations provided substrates capable of supporting probiotic growth. The moderate growth-promoting effect of the EF may be attributed to its naturally occurring carbohydrates together with polyphenolic constituents, which have previously been reported to modulate gut microbial composition and metabolism through selective microbial utilization and biotransformation [57,58].

Elderflower is rich in phenolic compounds, including chlorogenic acid and rutin, which have been associated with antioxidant activity and modulation of gut microbial metabolism [57,59]. In addition, residual soluble sugars naturally present in the extract may further contribute to bacterial proliferation.

The substantially greater stimulation observed for the inulin-containing formulations (EF + IN and EF + IN + PD) is consistent with the well-established prebiotic properties of inulin [58,60]. Inulin can be fermented by certain *Lactobacillus* species through the enzymatic degradation of inulin-derived fructans, generating fructose and shorter-chain fructooligosaccharides that can serve as carbon sources for bacterial growth [60]. The greater growth-promoting effect of these formulations therefore largely reflects the contribution of inulin as a readily fermentable substrate, while the elderflower extract may provide additional bioactive compounds that support bacterial growth. Although the stimulatory effect can be partly attributed to the prebiotic carriers, the formulations contained 80% elderflower extract and only 20% inulin or polydextrose, suggesting that both components contributed to the observed activity. The finding that EF + IN + PD did not consistently outperform EF + IN further indicates that the addition of polydextrose did not provide an additional growth-promoting benefit under the experimental conditions.

The observation that the EF + IN formulation containing only 20% inulin produced substantial growth stimulation, compared with pure inulin at the corresponding total concentration, which produced comparable or greater stimulation for some strains, suggests that the activity of EF + IN cannot be explained solely by the amount of inulin incorporated into the formulation and may reflect the combined contribution of the elderflower extract and the carrier matrix. Elderflower contains phenolic constituents chlorogenic acid derivatives and rutin, as well as naturally occurring soluble carbohydrates, which may contribute to its functional properties [57,59]. In addition, interactions between polyphenols and fermentable dietary fibers have been shown to modify the formation of phenolic metabolites and short-chain fatty acids. In an in vitro fermentation model, the presence of inulin affected the microbial transformation of rutin and altered the production of phenolic metabolites and SCFAs, demonstrating that fiber-polyphenol combinations may produce metabolic responses that differ from those observed with the individual components [61]. Recent in vivo evidence has further shown that polyphenols can modulate microbial responses within an inulin-rich dietary environment, with these effects depending on polyphenol structure and concentration [62]. In the present study, the FTIR results also indicated intermolecular interactions between elderflower constituents and the inulin matrix, suggesting that such matrix-polyphenol interactions may influence the physicochemical presentation and availability of EF constituents. Overall, these findings suggest that the enhanced functional activity of EF + IN may result from complementary contributions of the elderflower extract and inulin rather than from the carrier alone. One limitation of the present study is that it was not designed to formally evaluate combination effects using a specific dose–response synergy model; therefore, a synergistic interaction between elderflower polyphenols and inulin cannot be conclusively established. The observed effect should be considered a potentially complementary or enhanced formulation effect, warranting further investigation in dedicated combination studies.

The comparatively weaker activity of the EF + PD formulation is likewise consistent with the known fermentation characteristics of polydextrose. Complex and heterogeneous glucose polymer structure of polydextrose is generally fermented more slowly than inulin-type fructans and exhibits a weaker selective stimulatory effect toward probiotic bacteria [63]. This observation is further supported by the results obtained for the pure carrier materials, where inulin produced substantially greater growth stimulation than polydextrose alone.

The limited response of *S. boulardii* strains compared with *Lactobacillus* spp. suggests that the tested formulations exhibited selective prebiotic activity towards lactic acid bacteria. This is consistent with the concept of prebiotics as selectively utilized substrates that stimulate beneficial bacterial groups, particularly lactobacilli and bifidobacteria, while effects on yeasts are generally less pronounced [60,63].

Interestingly, the mixed probiotic culture consistently exhibited greater growth stimulation than the individual bacterial strains. This observation may reflect cooperative interactions among probiotic microorganisms, including metabolic cross-feeding, whereby metabolic intermediates produced by one species support the growth of other members of the community, resulting in more efficient substrate utilization and enhanced biomass production. Such synergistic interactions are well documented in mixed microbial communities inhabiting the gastrointestinal tract [64,65].

Overall, the results demonstrate that the spray-dried elderflower extract possesses growth-stimulating effects on probiotic microorganisms, which are notably enhanced by incorporation of inulin. Among the tested formulations, EF + IN and EF + IN + PD exhibited the strongest capacity to stimulate the growth of probiotic *Lactobacillus* strains, whereas polydextrose contributed only modestly under the conditions employed. These findings suggest that inulin-containing elderflower formulations may represent promising prebiotic ingredients capable of supporting the proliferation of beneficial intestinal bacteria.

### 3.5. In Vitro Release of Rutin and Chlorogenic Acid from Spray-Dried Powders

In vitro release of rutin and chlorogenic acid from spray-dried powders (EF, EF + PD, EF + IN, EF + PD + IN) was monitored in three media, with pH 1.2 relevant for the stomach in basal fasting conditions, pH 4.5 relevant for the stomach in the fed state, while pH 6.8 is used to simulate conditions in the small intestine [66].

According to the obtained results (Figure 6), rutin release from all samples was rapid under the applied experimental conditions. This observation is particularly significant considering the inherently low aqueous solubility of rutin, which is recognized as one of the major factors limiting its oral bioavailability [67]. According to previous reports, rutin is considered a poorly water-soluble flavonoid, with an aqueous solubility of approximately 0.09 mg/mL at room temperature [67]. Therefore, the solubility of rutin in the prepared powders was investigated. The results demonstrated a substantial increase in rutin solubility, reaching 4.37 ± 0.12 mg/mL for EF, 4.20 ± 0.21 mg/mL for EF + IN, 4.33 ± 0.18 mg/mL for EF + PD, and 4.32 ± 0.33 mg/mL for EF + IN + PD. The enhanced solubility can be attributed to the transformation of rutin from a crystalline to an amorphous state during the spray-drying process, as confirmed by DSC analysis. Compared with the crystalline form, amorphous materials possess higher free energy and a lower degree of molecular order, which promotes improved wettability, faster dissolution, and increased solubility. Similar observations have been reported for other spray-dried formulations with rutin, where amorphization significantly improved dissolution behavior relative to crystalline rutin [68,69].

Notably, the obtained results indicated that the spray-drying process increased rutin solubility by nearly 50-fold compared with its reported aqueous solubility. Such an improvement is particularly important given that the therapeutic potential of rutin, despite its well-established antioxidant, anti-inflammatory, anticancer, and vasoprotective activities, is often limited by poor dissolution and consequently reduced gastrointestinal absorption [67]. Therefore, the enhanced solubility observed in the developed spray-dried powders contributed to improved dissolution performance and could be significant for greater oral bioavailability of rutin.

Furthermore, it was evident that rutin was stable during in vitro release testing at pH 1.2 and 4.5, while during this test at pH 6.8, the content of rutin decreased slightly. The lower concentration of rutin in the dissolution medium at higher pH values may be attributed to its reduced chemical stability under neutral and alkaline conditions. Previous studies have shown that rutin remains relatively stable in acidic environments, whereas its degradation becomes more pronounced with increasing pH, resulting in the formation of quercetin and other phenolic degradation products [68].

Chlorogenic acid was rapidly released from all investigated samples at all tested pH values. This finding was expected since it is known that chlorogenic acid is soluble in water [70]. Nevertheless, a lower chlorogenic acid content was observed under near-neutral conditions, which may be attributed to its reduced stability and subsequent degradation under these conditions. Previous studies have demonstrated that chlorogenic acid degradation is both time- and pH-dependent, with the degradation rate increasing with increasing pH. Thus, chlorogenic acid is relatively stable under acidic conditions, whereas prolonged exposure to neutral and alkaline environments promotes its degradation [71].

## 4. Conclusions

As a rich source of bioactive compounds, *S. nigra* flower extract holds considerable potential for the prevention and management of diabetes, atherosclerosis, metabolic disorders, and the promotion of gut health. To enhance its stability, bioavailability, and functional properties, spray-dried microparticles were developed using the biocompatible carbohydrate polymers inulin and polydextrose, applied individually and in combination. The established encapsulation process yielded powders with promising technological and physicochemical properties, supporting their potential for further application in nutraceutical and pharmaceutical formulations.

The present results demonstrated that the carrier composition, rather than spray-drying conditions alone, substantially influenced particle-size characteristics, with inulin producing the smallest and most narrowly distributed particles and polydextrose yielding the broadest distribution. The incorporation of elderflower extract into carbohydrate matrices promoted the formation of an amorphous structure and enhanced thermal stability, likely through interactions between the carriers and bioactive compounds. Moreover, the amorphization process substantially increased the solubility of rutin and improved its dissolution behavior compared with crystalline rutin, demonstrating the potential of the developed formulations to enhance the physicochemical properties of poorly soluble bioactives.

The biological activity of elderflower extract was primarily associated with its high polyphenol content, with chlorogenic acid and rutin identified as the major constituents. Stability studies demonstrated that microencapsulation effectively preserved the phenolic profile of the powders during storage. In addition, the spray-dried formulations exhibited strong antioxidant activity, promising α-glucosidase inhibitory potential, and notable antimicrobial and prebiotic activities. Gram-positive bacteria were generally more susceptible to the elderflower formulations than Gram-negative strains, with the formulation containing inulin alone exhibiting the strongest antimicrobial activity, followed by the formulation containing both inulin and polydextrose. A similar trend was observed for the growth-promoting effects on probiotic microorganisms, with these formulations showing the greatest capacity to stimulate the growth of probiotic *Lactobacillus* strains.

The findings demonstrate that inulin and polydextrose can serve as dual-function carriers in elderflower extract microparticles, providing not only effective protection and preservation of bioactive compounds but also contributing to the enhancement of their functional properties, particularly those relevant to metabolic and gut health. The developed microencapsulates therefore represent promising multifunctional ingredients for incorporation into functional food, nutraceutical, and pharmaceutical formulations. However, further in vivo investigations are warranted to determine the systemic bioavailability and biological efficacy of the elderflower microencapsulates, focusing on their potential effects on gut microbiota modulation, as well as capacity to modulate key metabolic and diabetes-related biomarkers.

## Figures and Tables

**Figure 1:**
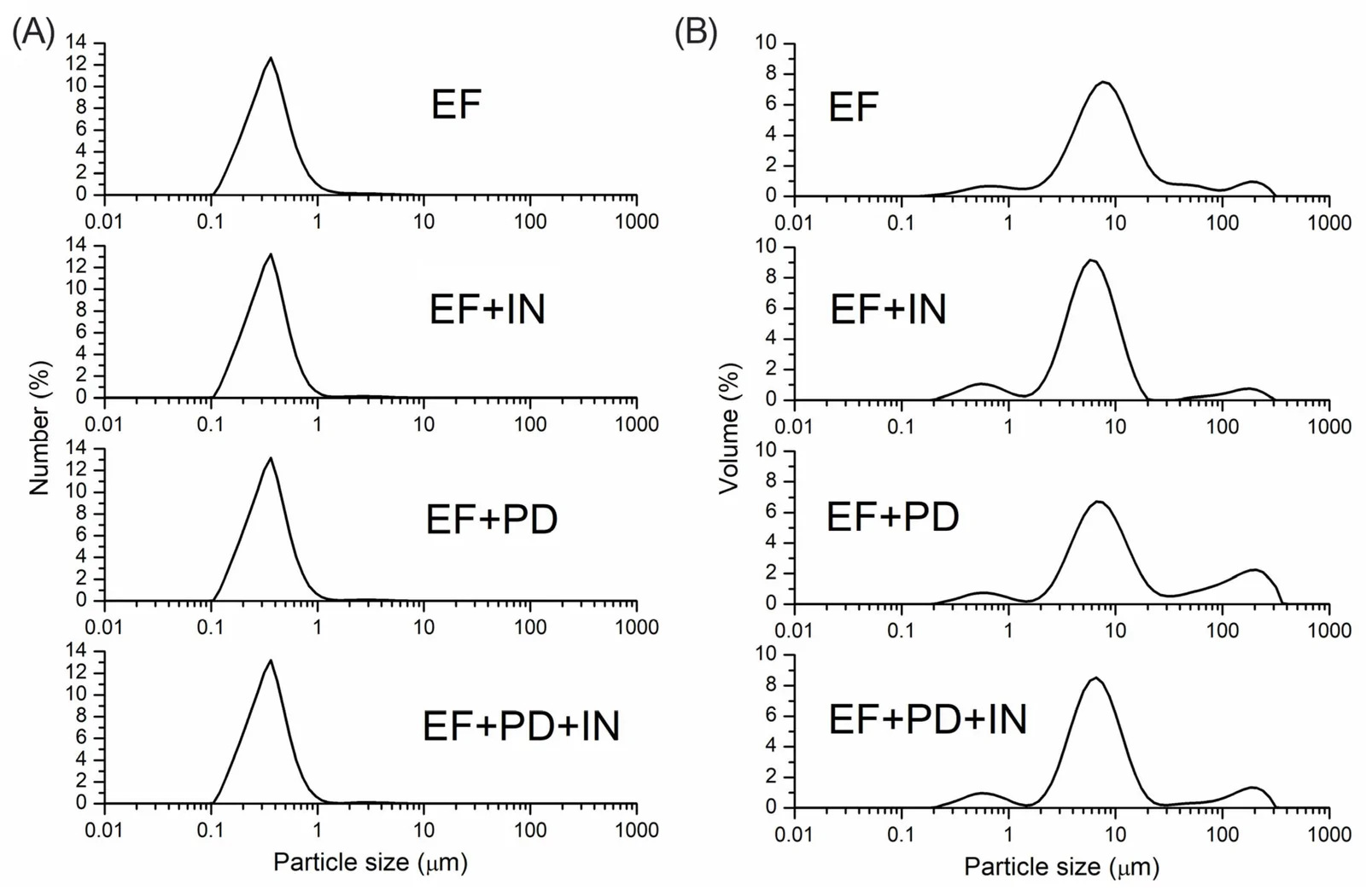
Particle size distribution of spray-dried elderflower extract (EF) and EF encapsulated powders within inulin (EF + IN), polydextrose (EF + PD) and carrier combinations (EF + PD + IN): (**A**) number-based and (**B**) volume-based analyses.

**Figure 2:**
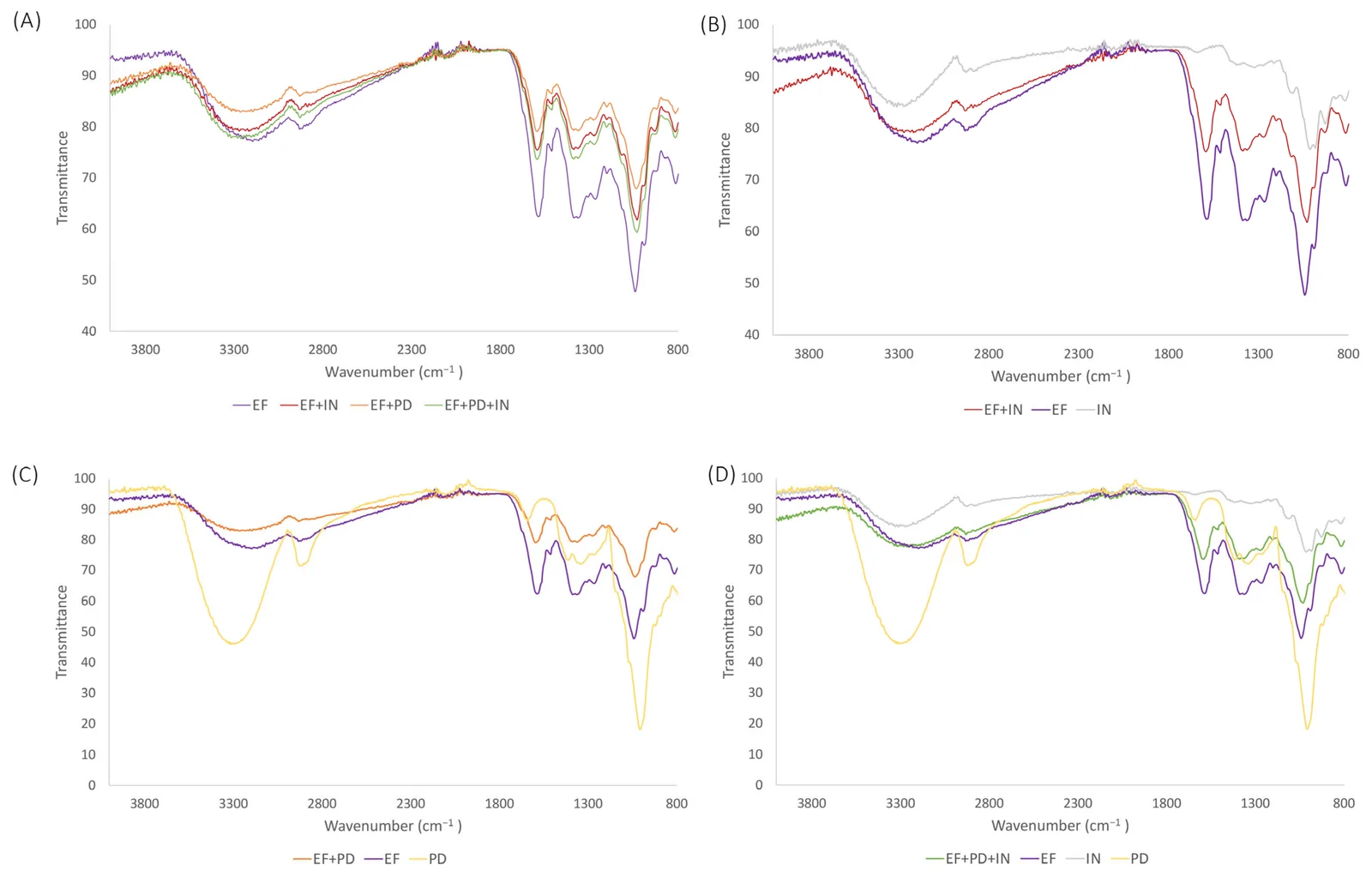
Fourier-transform infrared (FTIR) spectra of spray-dried elderflower powders ((EF): pure EF without a carrier; (EF + IN): EF containing 20% inulin; (EF + PD): EF containing 20% polydextrose; and (EF + IN + PD): EF containing a combination of carriers (10% IN and 10% PD)) and carriers ((IN): inulin; (PD): polydextrose). (**A**) FTIR spectra of EF, EF + IN, EF + PD, and EF + IN + PD samples. (**B**) FTIR spectra of EF, EF + IN, and IN samples. (**C**) FTIR spectra of EF, EF+PD, and PD samples. (**D**) FTIR spectra of EF, EF + IN + PD, IN and PD samples.

**Figure 3:**
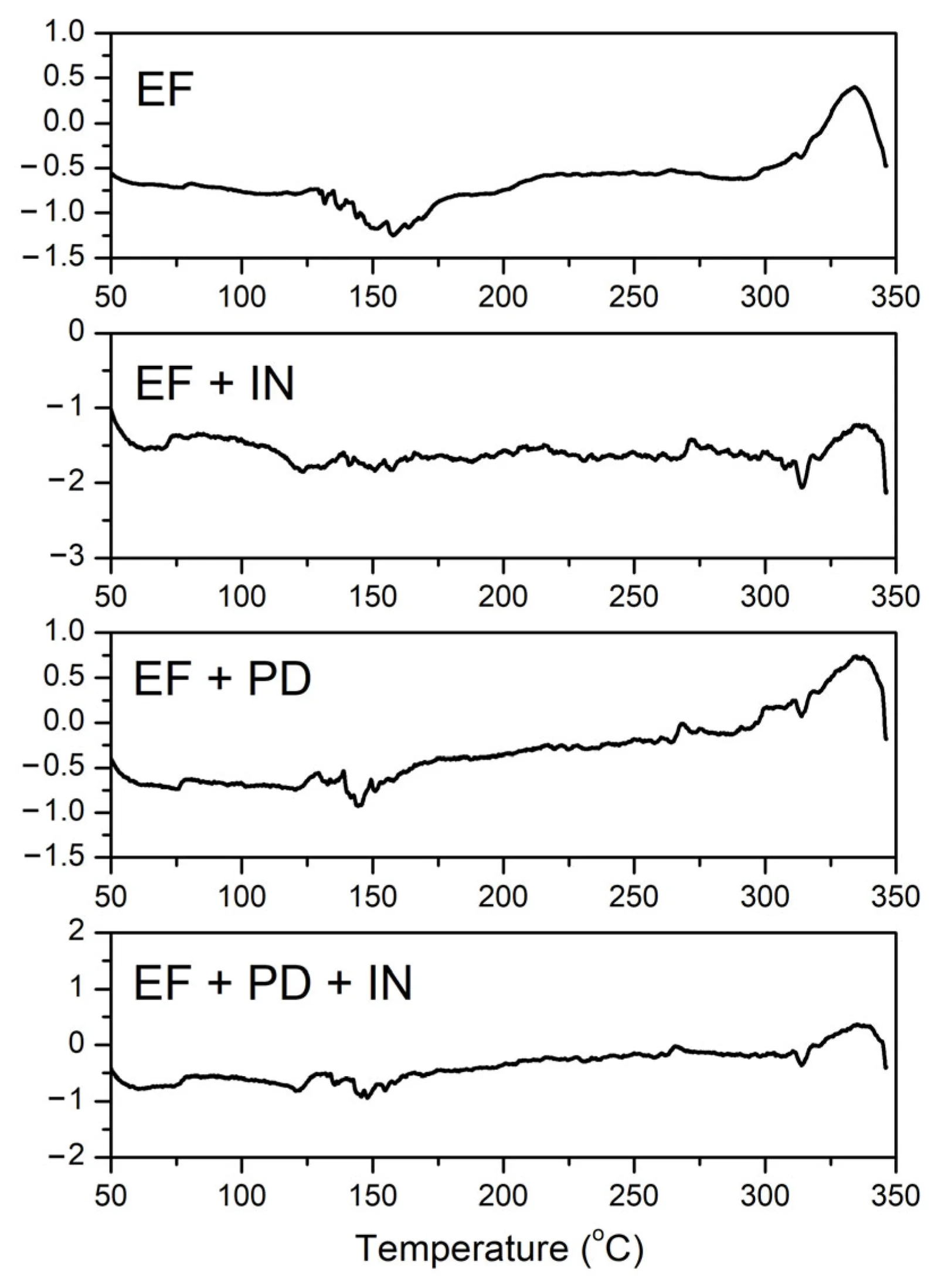
DSC thermograms of spray-dried elderflower extract (EF) and their respective encapsulated powders with carriers: inulin (EF + IN), polydextrose (PD), and carrier combination (EF + PD + IN).

**Figure 4:**
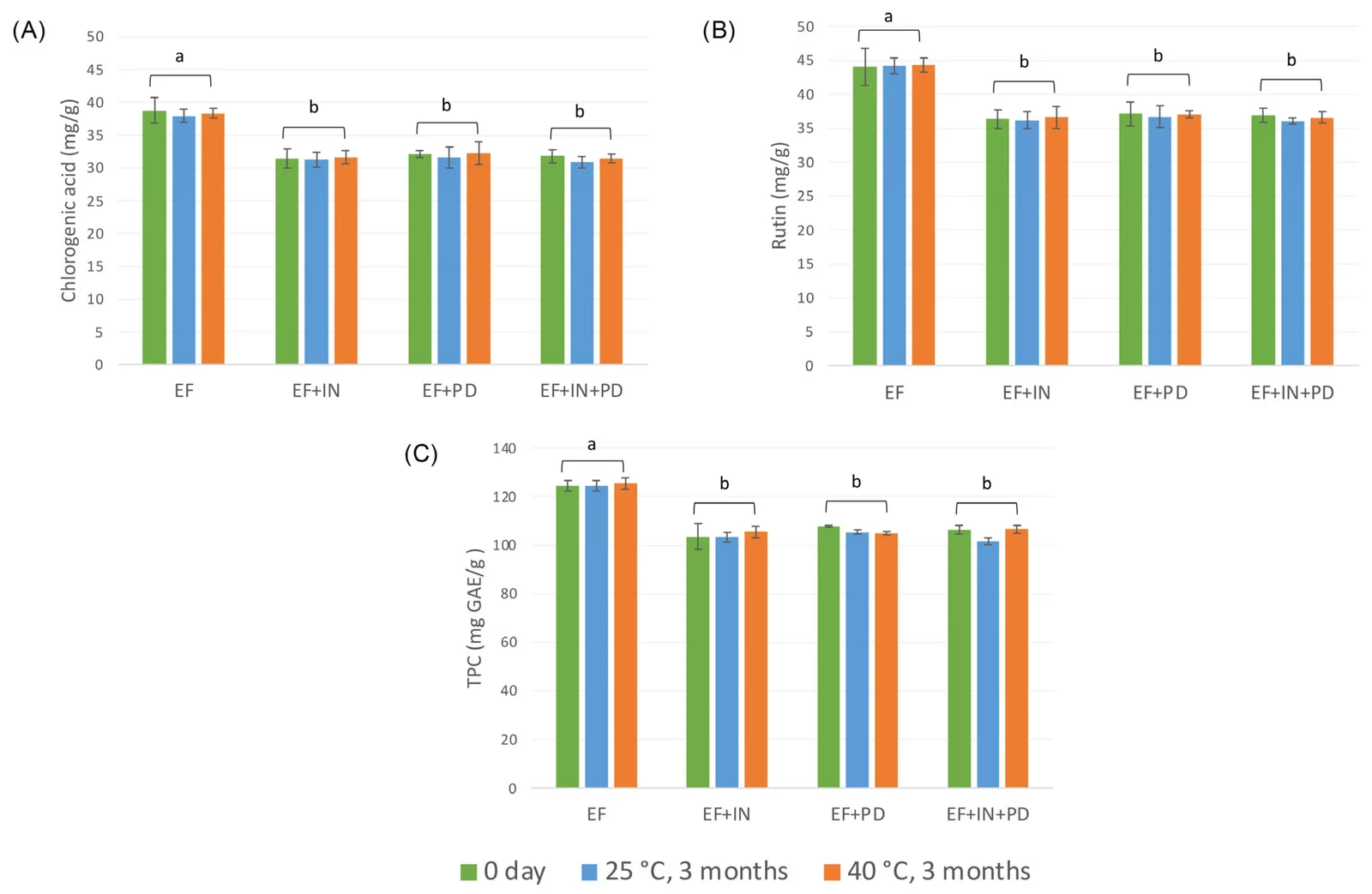
Stability analysis of spray-dried powders ((EF): pure EF without a carrier; (EF + IN): EF containing 20% inulin; (EF + PD): EF containing 20% polydextrose; and (EF + IN + PD): EF containing a combination of carriers (10% IN and 10% PD)) over a 30-day period. Means followed by different letters differ significantly, based on Tukey’s *post hoc* test at *p* < 0.05, *n* = 3. (**A**) Stability analysis for the chlorogenic acid content. (**B**) Stability analysis for the rutin content. (**C**) Stability analysis for the total phenolic content (TPC).

**Figure 5:**
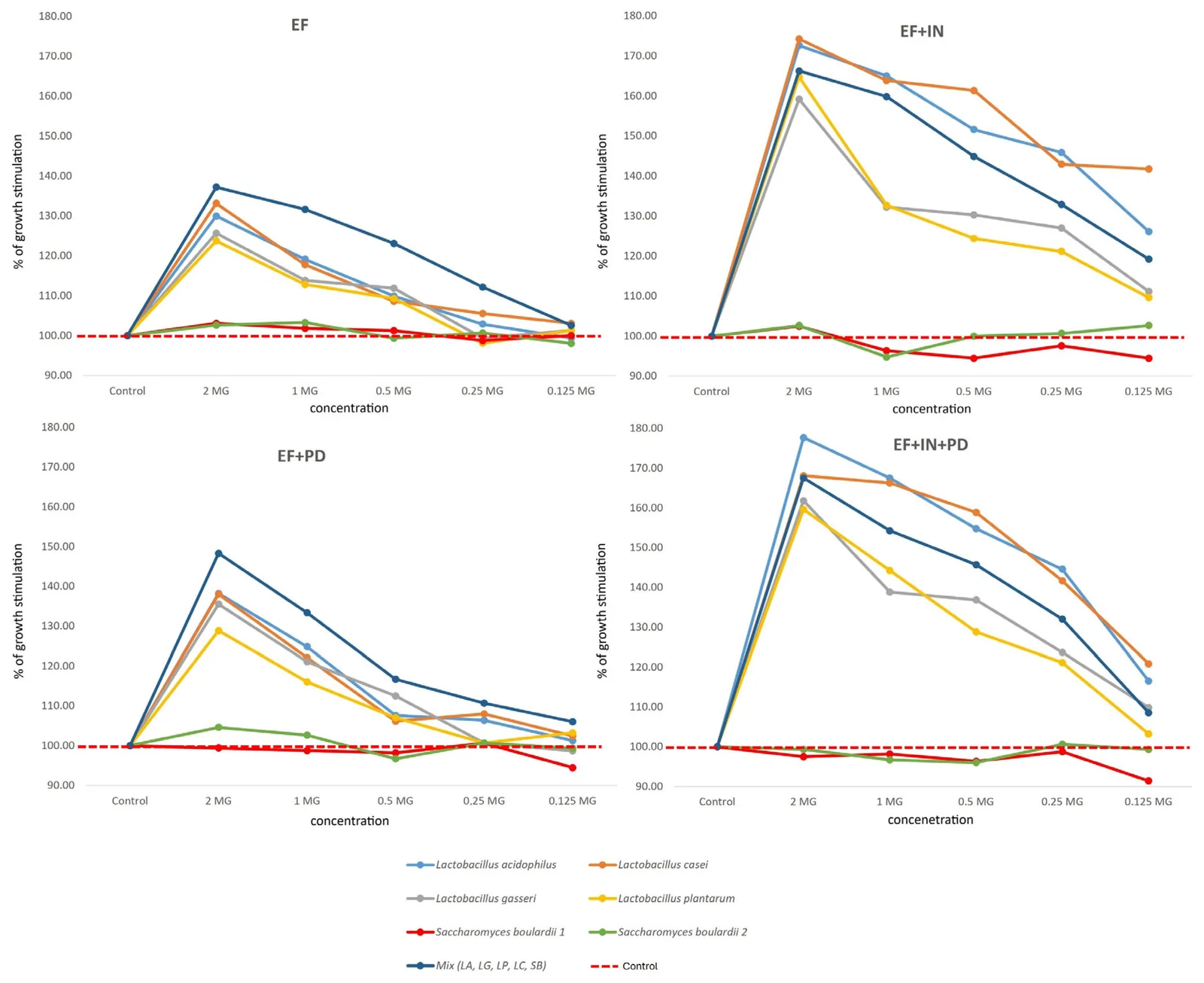
The effect of spray-dried elderflower extract on the growth of probiotic strains of microorganisms. Abbreviations: (EF): pure EF without a carrier; (EF + IN): EF containing 20% inulin; (EF + PD): EF containing 20% polydextrose; and (EF + IN + PD): EF containing a combination of carriers (10% IN and 10% PD). The results are presented as the percentage of growth relative to the untreated microorganisms (positive control, 100% growth).

**Figure 6:**
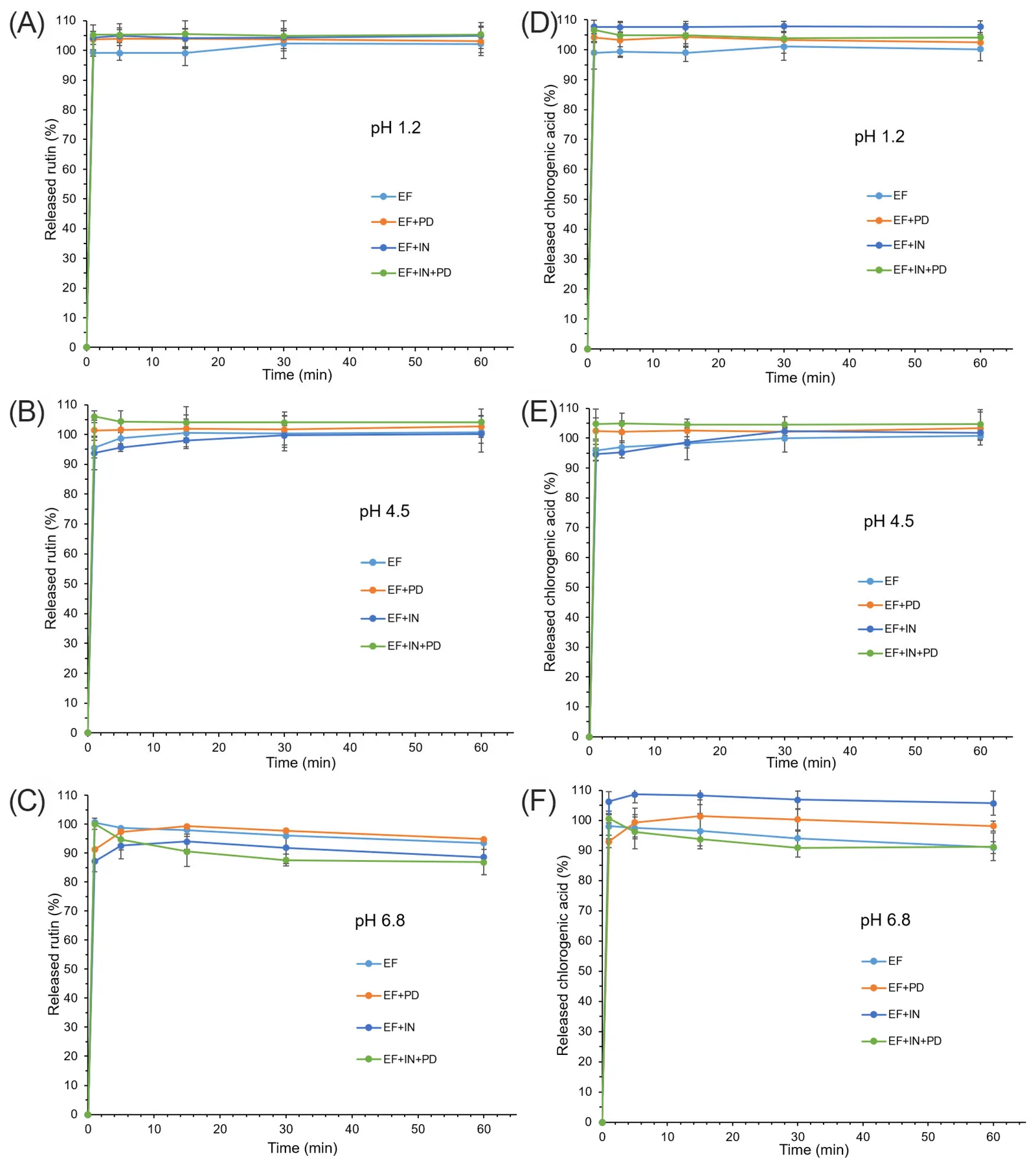
The release profile of rutin (**A**–**C**) and chlorogenic acid (**D**–**F**) from the investigated samples at pH 1.2, pH 4.5 and pH 6.8. Abbreviations: (EF): pure EF without a carrier; (EF + IN): EF containing 20% inulin; (EF + PD): EF containing 20% polydextrose; and (EF + IN + PD): EF containing a combination of carriers (10% IN and 10% PD).

**Table 1:** Technological properties of spray-dried elderflower powders prepared with different carriers.

Sample	Powder Yield (%)	Moisture Content	Bulk Density (g/mL)	Tapped Density (g/mL)	Carr Index (%)	Hausner Ratio
EF	27.68 ± 0.87 ^d^*	4.55 ± 0.23 ^a^	0.292 ± 0.01 ^a^	0.433 ± 0.01 ^a^	32.56 ± 1.25 ^b^	1.48 ± 0.07 ^a^
EF + IN	32.84 ± 0.71 ^c^	2.61 ± 0.08 ^c^	0.280 ± 0.01 ^a^	0.448 ± 0.02 ^a^	37.50 ± 1.24 ^a^	1.60 ± 0.06 ^a^
EF + PD	60.91 ± 2.09 ^a^	4.54 ± 0.10 ^a^	0.305 ± 0.01 ^a^	0.452 ± 0.01 ^a^	32.52 ± 1.31 ^b^	1.48 ± 0.06 ^a^
EF + IN + PD	50.26 ± 2.46 ^b^	3.33 ± 0.15 ^b^	0.288 ± 0.01 ^a^	0.443 ± 0.02 ^a^	34.99 ± 1.38 ^ab^	1.54 ± 0.06 ^a^

Abbreviations: (EF): pure EF without a carrier; (EF + IN): EF containing 20% inulin; (EF + PD): EF containing 20% polydextrose; and (EF + IN + PD): EF containing a combination of carriers (10% IN and 10% PD). * Means followed by different letters differ significantly, based on Tukey’s *post hoc* test at *p* < 0.05, *n* = 3.

**Table 2:** Particle size per number and volume of spray-dried elderflower extract (EF) and microencapsulates.

Particle Size per Number							
Samples	d_10_	d_50_	d_90_	Span	D [4,3]	D [3,2]	Uniformity
EF	0.17 ± 0.03 ^a^*	0.32 ± 0.05 ^a^	0.60 ± 0.07 ^a^	1.32 ± 0.12 ^a^	6.60 ± 0.50 ^a^	3.27 ± 0.40 ^a^	0.55 ± 0.06 ^a^
EF + IN	0.17 ± 0.02 ^a^	0.31 ± 0.02 ^a^	0.54 ± 0.05 ^a^	1.20 ± 0.16 ^a^	5.12 ± 0.30 ^b^	2.59 ± 0.32 ^a^	0.50 ± 0.04 ^a^
EF + PD	0.17 ± 0.02 ^a^	0.31 ± 0.03 ^a^	0.55 ± 0.06 ^a^	1.21 ± 0.15 ^a^	5.95 ± 0.45 ^ab^	2.87 ± 0.18 ^a^	0.50 ± 0.06 ^a^
EF + PD + IN	0.17 ± 0.02 ^a^	0.31 ± 0.05 ^a^	0.55 ± 0.04 ^a^	1.20 ± 0.09 ^a^	5.69 ± 0.39 ^ab^	2.78 ± 0.43 ^a^	0.49 ± 0.05 ^a^
**Particle Size per Volume**							
**Samples**	**d_10_**	**d_50_**	**d_90_**	**Span**	**D [4,3]**	**D [3,2]**	**Uniformity**
EF	2.36 ± 0.13 ^a^	7.38 ± 0.78 ^a^	35.51 ± 2.62 ^c^	4.49 ± 0.37 ^c^	19.40 ± 2.11 ^b^	3.59 ± 0.52 ^ab^	2.06 ± 0.27 ^b^
EF + IN	1.67 ± 0.09 ^b^	5.11 ± 0.30 ^b^	9.44 ± 1.23 ^d^	1.52 ± 0.17 ^c^	5.40 ± 0.67 ^c^	2.60 ± 0.24 ^b^	0.46 ± 0.05 ^c^
EF + PD	2.68 ± 0.42 ^a^	7.87 ± 1.18 ^a^	152.35 ± 10.98 ^a^	19.02 ± 1.99 ^a^	39.09 ± 3.00 ^a^	3.81 ± 0.46 ^a^	4.41 ± 0.34 ^a^
EF + PD + IN	2.28 ± 0.23 ^ab^	6.20 ± 0.82 ^ab^	52.45 ± 5.02 ^b^	8.10 ± 1.19 ^b^	21.31 ± 2.20 ^b^	3.08 ± 0.32 ^ab^	2.85 ± 0.43 ^b^

d_10_, d_50_, and d_90_ represent the sizes where 10%, 50%, and 90% of the particles are smaller than the remaining particles; * Means followed by different letters are significantly different according to the *post hoc* Tukey’s test at a level of *p* ≤ 0.05, *n* = 3. Abbreviations: (EF): pure EF without a carrier; (EF + IN): EF containing 20% inulin; (EF + PD): EF containing 20% polydextrose; and (EF + IN + PD): EF containing a combination of carriers (10% IN and 10% PD).

**Table 3:** Thermal transition parameters of spray-dried elderflower extract and spray-dried microencapsulates.

Sample	Transition	Temperature Range (°C)	Peak Maximum (°C)	ΔH (J/g)
EF	T1	129.05–184.36	157.86	74.08
	T2	184.59–209.78	196.25	4.82
EF + IN	T1	110.38–138.11	123.49	24.27
	T2	143.97–162.51	157.75	8.83
EF + PD	T1	129.47–138.94	132.86	4.74
	T2	138.88–168.80	144.41	30.57
EF + PD + IN	T1	100.89–128.72	120.55	19.36
	T2	133.85–166.33	148.10	33.86

Abbreviations: (EF): pure EF without a carrier; (EF + IN): EF containing 20% inulin; (EF + PD): EF containing 20% polydextrose; and (EF + IN + PD): EF containing a combination of carriers (10% IN and 10% PD).

**Table 4:** Phytochemical analysis of spray-dried powders.

Sample	Chlorogenic Acid (mg/g DW)	Rutin (mg/g DW)	TPC (mg GAE/g DW)
EF	38.75 ± 1.94 ^a^*	44.10 ± 2.71 ^a^	124.46 ± 2.26 ^a^
EF + IN	31.43 ± 1.51 ^b^	36.34 ± 1.34 ^b^	103.54 ± 5.19 ^b^
EF + PD	32.11 ± 0.57 ^b^	37.12 ± 1.80 ^b^	107.72 ± 0.34 ^b^
EF + IN + PD	31.80 ± 1.04 ^b^	36.96 ± 1.02 ^b^	106.36 ± 1.73 ^b^

Abbreviations: (EF): pure EF without a carrier; (EF + IN): EF containing 20% inulin; (EF + PD): EF containing 20% polydextrose; and (EF + IN + PD): EF containing a combination of carriers (10% IN and 10% PD). * Means followed by different letters differ significantly, based on Tukey’s *post hoc* test at *p* < 0.05, *n* = 3.

**Table 5:** Antioxidant and α-glucosidase and α-amylase inhibitory activities of spray-dried powders and reference standards.

Sample	DPPH IC_50_ (mg/mL)	α-Glucosidase IC_50_ (mg/mL)	α-Amylase (%)
EF	0.27 ± 0.02 ^b^*	4.18 ± 0.09 ^a^	3.36 ± 0.74 ^bc^
EF + IN	0.31 ± 0.02 ^b^	3.66 ± 0.05 ^b^	5.43 ± 0.57 ^a^
EF + PD	0.42 ± 0.03 ^a^	4.30 ± 0.05 ^a^	2.83 ± 0.25 ^c^
EF + IN + PD	0.42 ± 0.02 ^a^	3.74 ± 0.06 ^b^	4.64 ± 0.28 ^ab^
Chlorogenic acid	0.011 ± 0.001 ^c^	0.192 ± 0.008 ^cd^	0.688 ± 0.010 **
Rutin	0.008 ± 0.000 ^c^	0.114 ± 0.003 ^d^	0.053 ± 0.003 **
Acarbose	/	0.285 ± 0.019 ^c^	0.366 ± 0.018 **

Abbreviations: (EF): pure EF without a carrier; (EF + IN): EF containing 20% inulin; (EF + PD): EF containing 20% polydextrose; and (EF + IN + PD): EF containing a combination of carriers (10% IN and 10% PD). * Means followed by different letters differ significantly, based on Tukey’s *post hoc* test at *p* < 0.05, *n* = 3. ** α-amylase inhibition values for chlorogenic acid, rutin, acarbose were expressed as mg/mL.

**Table 6:** Results of antimicrobial activity for studied spray-dried powders.

Microbial Species	EF		EF + IN		EF + PD		EF + IN + PD	
Bacteria	MIC (mg/mL)	MBC (mg/mL)	MIC (mg/mL)	MBC (mg/mL)	MIC (mg/mL)	MBC (mg/mL)	MIC (mg/mL)	MBC (mg/mL)
*E. coli*	1.5	3.0	2.0	3.80	3.0	6.0	2.5	5.0
*P. aeruginosa*	2.0	4.0	2.5	5.0	4.0	8.0	3.2	6.4
*S. epidermidis*	0.5	1.25	0.8	1.8	1.5	2.5	1.0	2.0
*S. aureus*	0.5	1.0	0.8	1.6	1.2	3.2	0.9	1.8
*S. pyogenes*	0.6	1.2	1.0	2.0	2.0	4.0	1.5	2.6
Fungi	MIC (mg/mL)	MFC (mg/mL)	MIC (mg/mL)	MFC (mg/mL)	MIC (mg/mL)	MFC (mg/mL)	MIC (mg/mL)	MFC (mg/mL)
*C. albicans*	0.7	1.4	1.2	2.4	2.0	4.2	1.8	3.2

Abbreviations: (EF): pure EF without a carrier; (EF + IN): EF containing 20% inulin; (EF + PD): EF containing 20% polydextrose; and (EF + IN + PD): EF containing a combination of carriers (10% IN and 10% PD); (MIC): minimum inhibitory concentration; (MBC): minimum bactericidal concentration; (MFC): minimum fungicidal concentration.

**Table 7:** Growth-stimulation properties of spray-dried elderflower microparticles.

Extract/Microorganism			Concentration		
	2 mg/mL	1 mg/mL	0.5 mg/mL	0.25 mg/mL	0.125 mg/mL
EF					
*L. acidophilus*	129.9 ± 14.9	119.1 ± 16.1	109.9 ± 13.2	102.9 ± 13.8	99.4 ± 12.7
*L. casei*	133.1 ± 15.3	117.8 ± 15.9	108.6 ± 13.0	105.5 ± 14.1	103.1 ± 13.2
*L. gasseri*	125.7 ± 14.5	113.8 ± 15.4	111.8 ± 13.4	99.3 ± 13.3	101.3 ± 13.0
*L. plantarum*	123.7 ± 14.2	112.8 ± 15.2	109.3 ± 13.1	98.1 ± 13.1	101.3 ± 12.9
*S. boulardii* 1	103.1 ± 11.9	101.8 ± 13.8	101.2 ± 12.2	98.8 ± 13.2	100.0 ± 12.8
*S. boulardii* 2	102.6 ± 11.8	103.3 ± 13.9	99.3 ± 11.9	100.7 ± 13.5	98.0 ± 12.6
Mix (LA, LG, LP, LC, SB)	137.2 ± 15.8	131.6 ± 17.8	123.1 ± 14.8	112.2 ± 15.0	102.6 ± 13.1
EF + IN					
*L. acidophilus*	172.6 ± 20.7	165.0 ± 19.8	151.6 ± 18.2	145.9 ± 17.5	126.1 ± 10.1
*L. casei*	174.2 ± 20.9	163.8 ± 19.7	161.4 ± 19.4	142.9 ± 17.2	141.7 ± 11.3
*L. gasseri*	159.2 ± 19.1	132.2 ± 15.9	130.3 ± 15.6	127.0 ± 15.2	111.2 ± 8.9
*L. plantarum*	164.7 ± 19.8	132.7 ± 15.9	124.4 ± 14.9	121.2 ± 14.5	109.6 ± 8.8
*S. boulardii* 1	102.5 ± 12.3	96.3 ± 11.6	94.5 ± 11.4	97.6 ± 11.7	94.5 ± 7.6
*S. boulardii* 2	102.6 ± 12.3	94.7 ± 11.4	100.0 ± 12.0	100.7 ± 12.1	102.6 ± 8.2
Mix (LA, LG, LP, LC, SB)	166.2 ± 19.9	159.8 ± 19.2	144.9 ± 17.4	132.9 ± 16.0	119.2 ± 9.5
EF + PD					
*L. acidophilus*	138.2 ± 15.9	124.8 ± 16.9	107.6 ± 12.9	106.4 ± 14.3	101.3 ± 12.9
*L. casei*	138.0 ± 15.9	122.1 ± 16.5	106.1 ± 12.7	107.9 ± 14.5	102.5 ± 13.1
*L. gasseri*	135.5 ± 15.6	121.1 ± 16.3	112.5 ± 13.5	100.7 ± 13.5	98.7 ± 12.6
*L. plantarum*	128.8 ± 14.8	116.0 ± 15.7	107.1 ± 12.9	100.6 ± 13.5	103.2 ± 13.2
*S. boulardii* 1	99.4 ± 11.4	98.8 ± 13.3	98.2 ± 11.8	100.6 ± 13.5	94.5 ± 12.1
*S. boulardii* 2	104.6 ± 12.0	102.6 ± 13.9	96.7 ± 11.6	100.7 ± 13.5	99.3 ± 12.7
Mix (LA, LG, LP, LC, SB)	148.3 ± 17.1	133.3 ± 18.0	116.7 ± 14.0	110.7 ± 14.8	105.9 ± 13.6
EF + IN + PD					
*L. acidophilus*	177.7 ± 21.3	167.5 ± 20.1	154.8 ± 18.6	144.6 ± 17.4	116.6 ± 10.3
*L. casei*	168.1 ± 20.2	166.3 ± 19.9	158.9 ± 19.1	141.7 ± 17.0	120.9 ± 10.6
*L. gasseri*	161.8 ± 19.4	138.8 ± 16.7	136.8 ± 16.4	123.7 ± 14.8	109.9 ± 9.7
*L. plantarum*	159.6 ± 19.2	144.2 ± 17.3	128.9 ± 15.5	121.2 ± 14.5	103.2 ± 9.1
*S. boulardii* 1	97.6 ± 11.7	98.2 ± 11.8	96.3 ± 11.6	98.8 ± 11.9	91.4 ± 8.0
*S. boulardii* 2	99.4 ± 11.9	96.7 ± 11.6	96.1 ± 11.5	100.7 ± 12.1	99.3 ± 8.7
Mix (LA, LG, LP, LC, SB)	167.5 ± 20.1	154.3 ± 18.5	145.7 ± 17.5	132.1 ± 15.9	108.6 ± 9.6

Results presented as % of growth stimulation relative to the positive control, expressed as mean ± SD, (*n* = 3), after cultivation with extracts in the range of 0.125–2 mg/mL. Abbreviations: (EF): pure EF without a carrier; (EF + IN): EF containing 20% inulin; (EF + PD): EF containing 20% polydextrose; and (EF + IN + PD): EF containing a combination of carriers (10% IN and 10% PD); (IN): inulin; (PD): polydextrose.

## Data Availability

Data is contained within the article.
